# Optomechanically Actuated Hydrogel Platform for Cell Stimulation with Spatial and Temporal Resolution

**DOI:** 10.1021/acsbiomaterials.3c00516

**Published:** 2023-08-21

**Authors:** Allison N. Ramey-Ward, Yixiao Dong, Jin Yang, Hiroaki Ogasawara, Elizabeth C. Bremer-Sai, Olga Brazhkina, Christian Franck, Michael Davis, Khalid Salaita

**Affiliations:** † Wallace H. Coulter Department of Biomedical Engineering, Georgia Institute of Technology & Emory University, Atlanta, Georgia 30322, United States; ‡ Department of Chemistry, Emory University, Atlanta, Georgia 30322, United States; § Department of Mechanical Engineering, University of Wisconsin − Madison, Madison, Wisconsin 53706, United States

**Keywords:** optomechanical actuator, hydrogel, myogenesis, mechanobiology

## Abstract

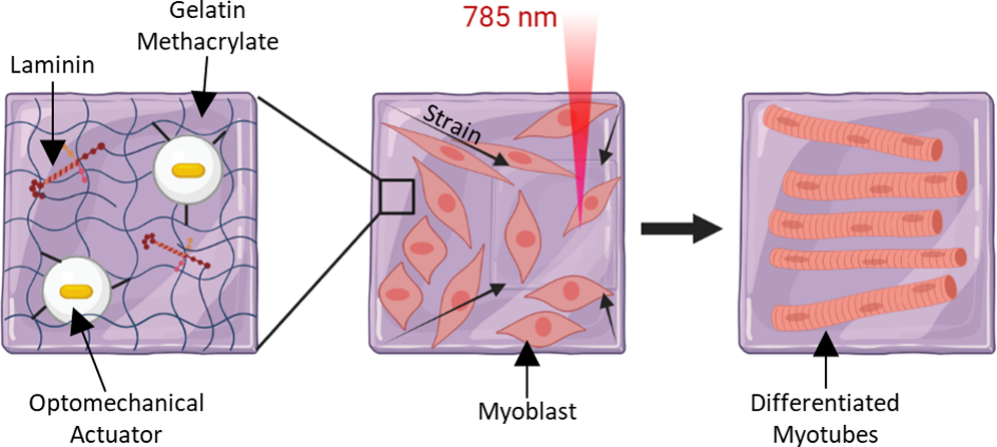

Cells exist in the body in mechanically dynamic environments, yet the vast majority of *in vitro* cell culture is conducted on static materials such as plastic dishes and gels. To address this limitation, we report an approach to transition widely used hydrogels into mechanically active substrates by doping optomechanical actuator (OMA) nanoparticles within the polymer matrix. OMAs are composed of gold nanorods surrounded by a thermoresponsive polymer shell that rapidly collapses upon near-infrared (NIR) illumination. As a proof of concept, we crosslinked OMAs into laminin-gelatin hydrogels, generating up to 5 μm deformations triggered by NIR pulsing. This response was tunable by NIR intensity and OMA density within the gel and is generalizable to other hydrogel materials. Hydrogel mechanical stimulation enhanced myogenesis in C2C12 myoblasts as evidenced by ERK signaling, myocyte fusion, and sarcomeric myosin expression. We also demonstrate rescued differentiation in a chronic inflammation model as a result of mechanical stimulation. This work establishes OMA-actuated biomaterials as a powerful tool for *in vitro* mechanical manipulation with broad applications in the field of mechanobiology.

## Introduction

Cell culture is an important technique in biology, allowing researchers to investigate the specifics of cell and tissue processes *in vitro* without costly and invasive *in vivo* and clinical studies. This ability to study and manipulate cells in a controlled environment has led to innumerable advances in the biomedical sciences. However, work *in vitro* (literally “in glass”) rarely replicates the true environment of the body. Indeed, 2D cell culture on glass or polystyrene substrates can greatly skew cell behaviors,^1,2^ which limits not only the understanding of physiology but also the effective development of therapeutics.^3^

One important challenge in these systems is that most cell culture substrates are stiff and mechanically static, whereas cells in the body, from blood to bone, all experience a dynamic physical and mechanical environment with repetitively applied strains that are sensed and transduced,^4−7^ by many types of mechanosensitive receptors such as integrins.^8,9^ Cell mechanosensitivity is widely demonstrated: for example, changing culture substrate mechanics can profoundly influence cell biology.^10−12^ From this modulation of mechanical stiffness, the next step in understanding how cells behave in the body is to replicate the repetitive and dynamic strains applied of their native environment.

To probe this growing area of mechanobiology, different types of mechanically active hydrogels have been reported in the literature. The first class of these materials are gels that can be controllably deformed through bulk manipulation. For example, cyclic strain bioreactors (CSBs) use a soft material substrate attached to actuators that can apply strain at a certain magnitude, rate, and frequency to layers of attached cells.^13−16^ These systems combine softer substrates and mechanical dynamics to enhance cellular processes such as differentiation,^17,18^ creating a biomimetic culture environment.^19,20^ Yet these instruments lack the ability to stimulate cells with spatial control, which is important as the physiological environment is not mechanically homogeneous.^21,22^ This inhomogeneous mechanical microenvironment has a profound impact on cell responses,^23−25^ leading to the development of the second class of active gels: those that are locally deformable. This second class includes patterned responsive gels, such as the HAIRS system developed by Sutton et al. composed of micropillars patterned in a bulk temperature-responsive gel.^26^

Despite the advantages of locally deformable gels, these approaches either allow only for application of forces to cells via artificially formed “islands” of adhesions at the tips of micropillars or require patterning of the active gel material. Both are undesirable as these gels are limited in the composition of the material, often requiring sub-physiological cell culture temperatures to control actuation.

In this work, we present a novel light-driven mechanically active hydrogel cell culture platform that can be applied to virtually any type of hydrogel. Previously, our group reported the optomechanical actuator (OMA), a composite nanoparticle composed of a gold nanorod (AuNR) core encapsulated by a poly-*N*-isopropyl methacrylamide (pNIPMAm) shell with a transition temperature of 42 °C.^27^ With these nanoactuators, we have demonstrated mechanically driven control of mechanotransduction in multiple biological models and cell types.^27,28^ Herein, we incorporate OMAs into polymeric hydrogel matrices to create a modular platform for remotely actuatable cell culture substrates.

This approach, which allows remotely applied, micron-scale deformations to cells using NIR illumination, provides several distinct advantages, including simplifying the required instrumentation compared to other mechanically active systems such as CSBs. Moreover, we still retain the ability to directly actuate whole cells or groups of cells at once, which is not possible with single-cell techniques such as atomic force microscopy, nor with OMAs alone. Furthermore, the deformation of the reported hydrogel system, and the area actuated, is tunable by altering the NIR laser intensity or the OMA content of the hydrogel. We also show this method of OMA incorporation can be applied to multiple types of hydrogel biomaterials, and we demonstrate modularity by including OMAs in gelatin methacrylate (GelMA), collagen, and poly(ethylene glycol) (PEG). Importantly, OMAs can be combined with 3D bioprinting techniques to further improve spatial control.

OMA-doped hydrogels provide a novel approach to studying cell biology, opening paths to investigate the complex mechanically dynamic environments of native tissue *in vitro*. As a proof-of-concept demonstration, we use OMA-stimulated gels to control mechanotransductive pathways in the differentiation of C2C12 skeletal muscle myoblasts. Skeletal muscle cells in the body reside in a very mechanically dynamic 3D environment. Because of this, many signaling pathways involved in myogenesis, including the extracellular signal-related kinase (ERK) pathway as well as the Yes-associated protein (YAP) pathway, are highly mechanosensitive in their activation.^25,28^

We show our mechanically active hydrogel impacts multiple areas of muscle cell biology. Mechanically stimulated C2C12 myoblasts demonstrated mechanosensitive responses, evidenced by directional morphological changes and YAP nuclear localization. These cells also show enhanced myogenic differentiation and fusion, and hydrogel mechanical stimulation resulted in ERK activation profiles consistent with trends of myoblast maturation. Finally, we found mechanical stimulation rescued C2C12 cells from the inflammatory phenotype when treated with TNFα. As such, we demonstrate our hydrogel biomaterial as a novel platform for studying cell biology and pathology in a more biomimetic environment *in vitro*.

## Results

### Hydrogel Formation and Bulk Characterization

OMAs were synthesized following our prior published protocols.^27−30^ Most OMA particles incorporated the 120 × 20 nm^2^ AuNR core (Figure S1a) and displayed an average dehydrated diameter of 340 ± 40 nm (Figure S1b). OMAs had a hydrodynamic diameter in solution of 560 ± 130 nm at room temperature (RT) and 280 ± 30 nm above 40 °C (Figure S1c).

Because the mechanical action of OMAs is dependent on the thermoresponsivity of the pNIPMAm shell, we tested to ensure that OMAs incorporated into a hydrogel matrix would continue to display thermoresponsive properties. Actuating hydrogels with varying OMA contents were heated above the reported LCST of NIPMAm (42 °C) and change in gel size was quantified. There was an OMA content-dependent decrease in size due to heating (Figure S1d).

To facilitate OMA incorporation into GelMA hydrogels, an azido-functionalized methacrylate crosslinker was synthesized (Figure S2a–c) and coupled to alkyne-modified OMAs by a copper-catalyzed click reaction. After conjugation of azido-MA to the alkyne-OMAs, methacrylated OMAs (MA-OMAs) were coupled to fluorescein-*O*-methacrylate (1000× excess) to validate MA incorporation (Figure S2d). MA-OMAs showed significantly higher fluorescein fluorescence compared to nonmethacrylated OMAs (Figure S2e,f).

**Figure 1:**
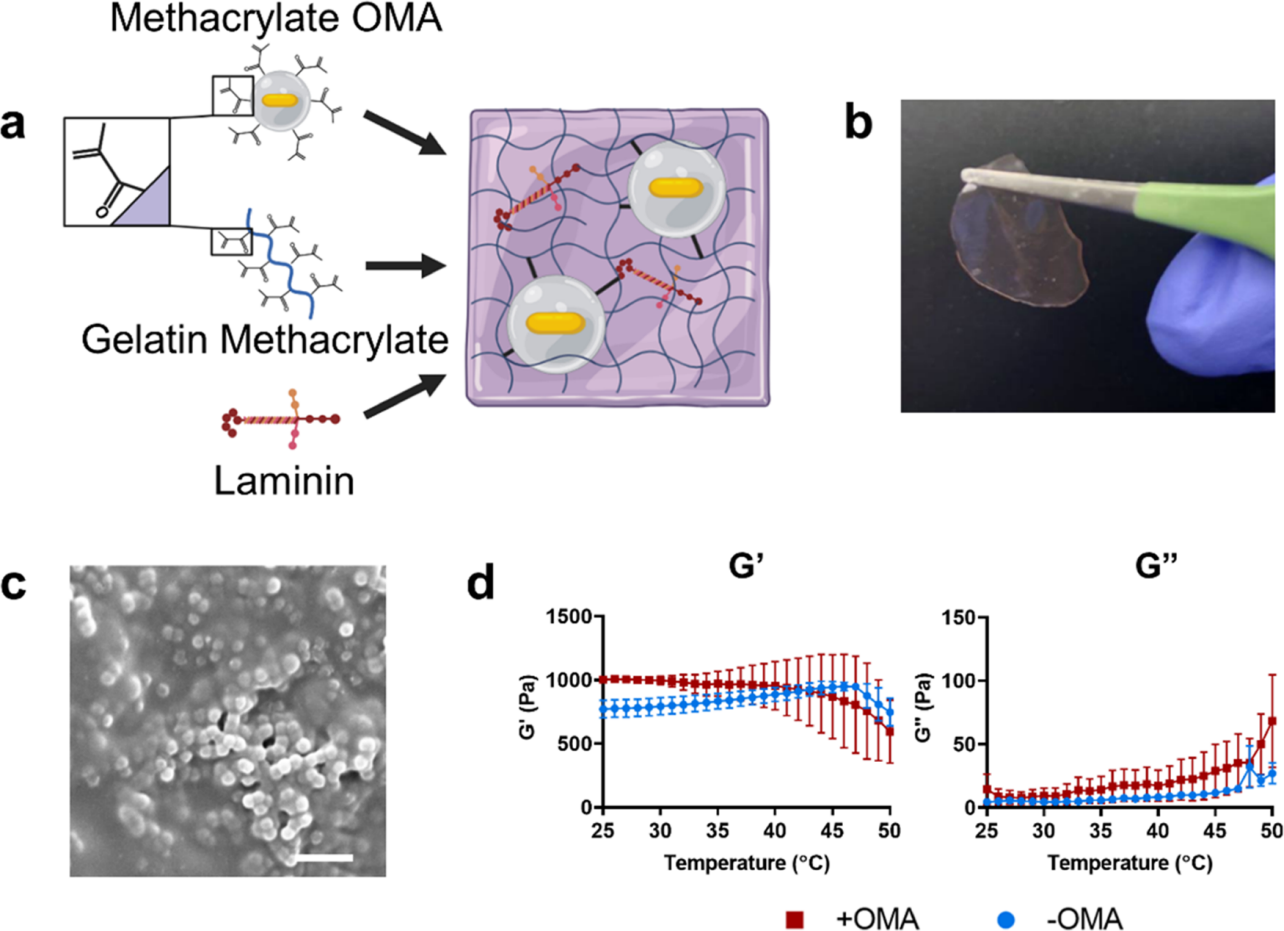
Characterization of actuating hydrogels. (a) Schematic of hydrogel composition. Methacrylate-modified OMAs, gelatin methacrylate, and mouse laminin were combined and polymerized to form actuating hydrogels. Schematic not drawn to scale. (b) Photograph of actuating hydrogel. (c) Scanning electron microscopy image of the actuating hydrogel surface, showing OMAs on the gel surface and in the gel bulk. Scale bar: 2 μm. (d) Temperature-controlled rheometry comparing the response of hydrogels upon incorporation of OMAs: (left) storage modulus (*G*′), (right) loss modulus (*G*″). Graph shows mean and SEM for 2 repeated measurements on representative gels.

Actuating hydrogels were formed by combining 14.5 nM OMAs with a 10% w/v GelMA solution in a 1:4 volume ratio, along with mouse laminin and photocrosslinking initiators under white light as described in the Materials and Methods section (Figure 1a). The hydrogels were flat as examined by the naked eye, with a slight red coloration due to the AuNR incorporation (Figure 1b). Imaging dehydrated gels with SEM confirmed the homogeneous incorporation of ∼400 nm spherical structures distributed throughout the gel (Figure 1c).

To investigate if gels would degrade during storage or cell culture, GelMA constructs with varying OMA contents were incubated for 3 weeks in C2C12 growth media at 4 or 37 °C. While gels containing 10% OMAs degraded almost completely within 1 week at 37 °C, higher OMA concentrations were more stable at 37 °C and even exhibited swelling at 4 °C (Figure S3).

No statistically significant change in gel behavior was observed above 42 °C upon incorporation of OMAs in either storage modulus (*G*′) or loss modulus (*G*″), though OMA-containing hydrogels were slightly stiffer at RT (Figures 1d and S4a–d). When cooled from 50 °C to RT, OMA-containing gels also exhibited a slightly different pattern of *G*′ response compared to control GelMA with a slight stiffening of the gel from 50 to 45 °C before returning to its baseline stiffness (Figure S4e,f). *G*″ exhibited similar behavior in all tested conditions (Figure S4g,h), increasing in its viscous behavior at higher temperatures as expected when a hydrogel approaches its melting point (>60 °C).

### Actuating Hydrogel NIR Responsivity Is a Function of Illumination Power and OMA Content

**Figure 2:**
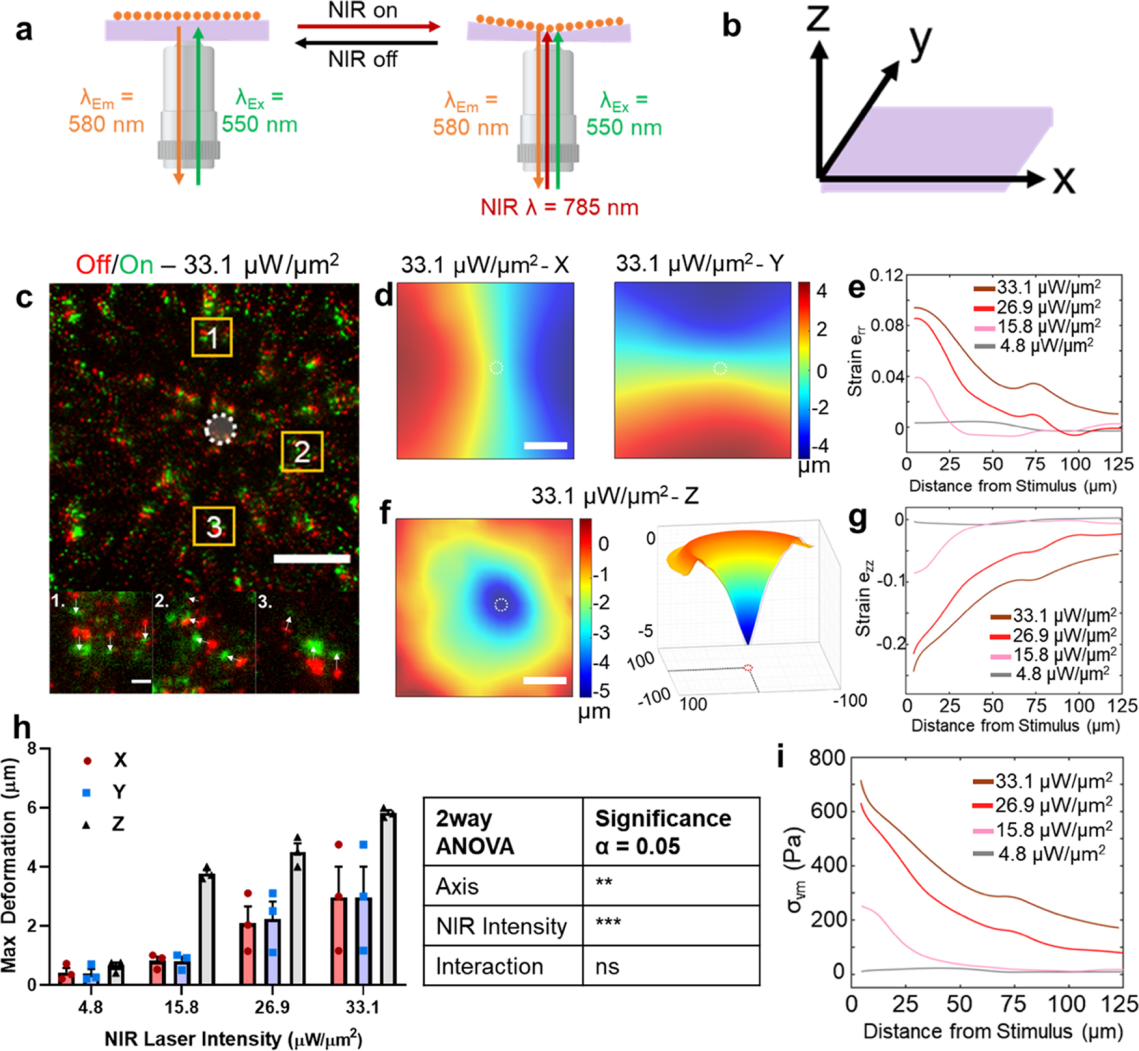
NIR Responsivity of actuating hydrogels. (a) Schematic of gel responsivity of GelMA hydrogels (purple) containing 20% v/v OMAs. When illuminated with NIR light (red), gels contract toward the laser stimulus, as evidenced by the movement of fluorescent markers on the surface of the gel (orange). (b) Reference axes for discussion of gel deformation, where *XY* is parallel with the undeformed gel surface and *Z* is the dimension representing the thickness of the gel. (c) Overlay of fluorescence images showing displacement of fluorescent beads on the surface of actuating gels between the unilluminated gel (“off”, red) and the gel illuminated with NIR light at 33.1 μW/μm^2^ (“on”, green). The white circle indicates NIR illumination, scale bar: 50 μm. Orange boxes indicate outsets, showing particle movement between NIR illumination conditions (white arrows). Scale bar: 5 μm. (d) Representative deformation of the actuating hydrogels in the *x* (left) and *y* (right) dimensions, calculated by ALDVC. Scale bar: 50 μm. The white circle indicates NIR stimulus location. (e) Radial strain plot (*e*_rr_) shows strain dependence on laser power and distance from stimulus. (f) (Left) *Z* deformation, calculated by ALDVC, in actuating hydrogels shows decreasing deformation as a function of distance from the stimulus location (white circle). Scale bar: 50 μm. (Right) 3D plot of *z* deformation visualizing the contraction of the gel surface toward the underlying glass substrate. (g) Plot showing gel strain in the *z* dimension (*e*_zz_, right), at multiple NIR power densities. (h) Graph showing mean ± SEM of the maximum deformation observed in each dimension at multiple laser intensities. Significance calculated by two-way ANOVA for *n* = 3 independently prepared gels. **p < 0.01, ***p < 0.001. (i) von Mises stress calculated within actuating gels as a function of distance from the NIR stimulus location at multiple NIR power densities.

To study photothermal responsivity, we characterized the response of actuating hydrogels driven by NIR triggering. Hydrogel surfaces were modified with fluorescent beads to serve as visual markers (Figure 2a). Z-stack acquisitions through the volume of the gel with and without NIR illumination were then processed using the augmented Lagrangian digital volume correlation (ALDVC) method.^31^ Hydrogels responded rapidly to NIR illumination—contracting in tens to hundreds of ms (Video S1) and showed contraction in both the XY plane as well as the *Z* direction (Figure 2a,b). Gels containing MA-OMAs showed more deformation under NIR illumination than those containing nonmethacrylated OMAs (Figure S5a,b). Because of this, all OMAs referred to after this point in this work are MA-OMAs unless specified. Gels containing no OMAs showed no response to NIR (Figure S5c,d).

*XY* deformation was symmetric around the area of NIR illumination, moving inward toward the laser as expected for a contractile system (Figure 2c,d). Gel deformation in *Z* demonstrated an expected depression in the gel centered around the NIR illumination (Figure 2f). The strain was greatest near the laser illumination spot and decreased as a function of distance in all tested laser power densities. In contrast, the magnitude of the deformation, stress, and strain decreases with decreasing laser power (Figures 2e,g–i and S6).

We also observed that the maximum XY deformation was located some distance from the NIR stimulus (Figures 2e, S6a–c, and S7a–c). Near the stimulus, the higher *Z* deformation dominated the observed signal, resulting in little to no detected motion in the plane of the image. Farther from the NIR stimulus, particle lateral motion was more significant, causing an increase in deformation distal to the stimulus. Furthermore, the mechanical response within the gel was demonstrated to be distance-dependent by the calculation of strain and stress within the gel in each dimension, assuming a Poisson’s ratio of *ν* = 0.495 (Figure 2e,g,i).

To investigate the tunability of the gel responsiveness to NIR illumination, we prepared gels with varying OMA contents. Deformation decreased with decreasing OMA content and again showed greater *Z* deformation than *XY* deformation (Figure S7). Because we wanted to characterize the maximum responsivity of the actuating hydrogel, all subsequent experiments utilized the 33.1 μw/μm^2^ NIR illumination power density to stimulate gels containing 20% OMAs unless specified.

### Suitability of Actuating Hydrogels for Cell Culture

Having characterized the responsivity of the actuating gels, we next investigated the suitability of these gels for cell culture. While the total number of attached cells was not affected by the inclusion of laminin into actuating hydrogels (Figure S8a,b), their spreading area was significantly increased (Figure S8a,c).

Surface heating of actuating hydrogels was visualized by submerging gels in a rhodamine solution imaging under NIR stimulation. NIR intensity had little effect on heating at the gel surface (Figure S9a,b) where cells are cultured, but the stimulation duty cycle caused a significant change in heating, up to 3 °C, which was transient and reversible at 50% duty cycle (Figure S9c,d). Finally, a cell viability assay showed no cellular uptake of DAPI after stimulation with NIR stimulation cycle used in our study (Figure S9e).

### C2C12 Myoblasts Extend in the Direction of NIR Stimulus

**Figure 3:**
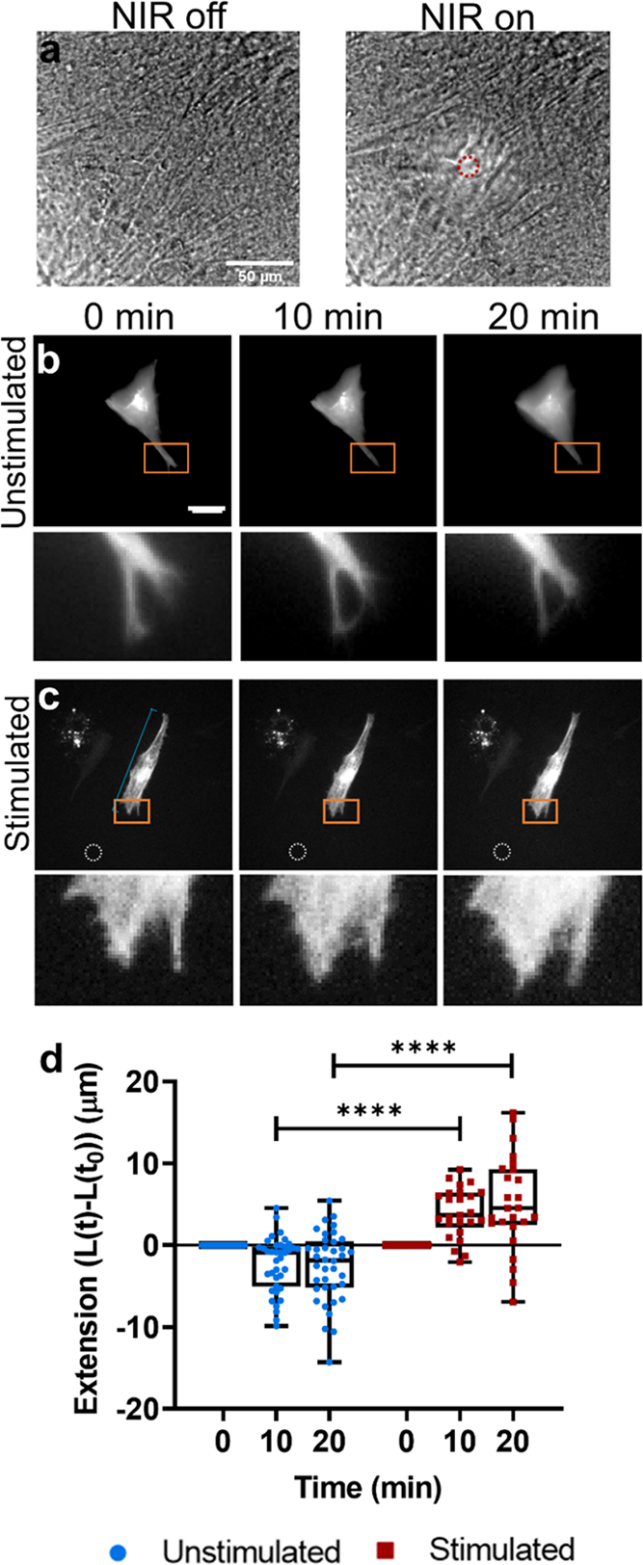
Morphological response of cells on actuating hydrogels. (a) Bright-field microscopy images showing deformation of a dense C2C12 monolayer under 33.1 μW/μm^2^ NIR illumination (red circle). (b, c) Representative images of C2C12 myoblast transfected with mCherry LifeAct on actuating gels, either (b) unstimulated or (c) stimulated with NIR at 1 Hz (50% duty cycle) at 33.1 μW/μm^2^ NIR illumination (white circle) for 20 min. Scale bar: 20 μm. Outsets (orange boxes) of cell edges show the extension of cells in the direction of NIR stimulation over the observed time period. (d) Box plot showing the range of cell extension as a function of time in unstimulated (blue) and stimulated (red) cells. ****p < 0.0001 by mixed-effects model with Sidak’s multiple comparisons for at least 25 cells on *n* = 3 independently prepared hydrogels.

We first investigated the myogenic effects of the actuating gel system morphologically by quantifying myoblast elongation. C2C12s plated on actuating hydrogel surfaces exhibited extension in the direction of NIR stimulation after as little as 10 min of actuation at 1 Hz, whereas unstimulated cells showed no extension (Figure 3).

We controlled for the effects of AuNR photothermal heating by C2C12s on 10% GelMA hydrogels containing an equal concentration of AuNRs as OMA experiments, and stimulated for the same amount of time. These gels underwent a marginal amount of deformation in the *Z* dimension (Figure S10a,b), nearly 75% less than OMA-containing gels (Figure 2c–i). Cells stimulated on AuNR gels did not behave differently than those on unstimulated gels (Figure S10c,d).

### Myoblast Nuclear YAP Is Enhanced on NIR-Stimulated Hydrogels

**Figure 4:**
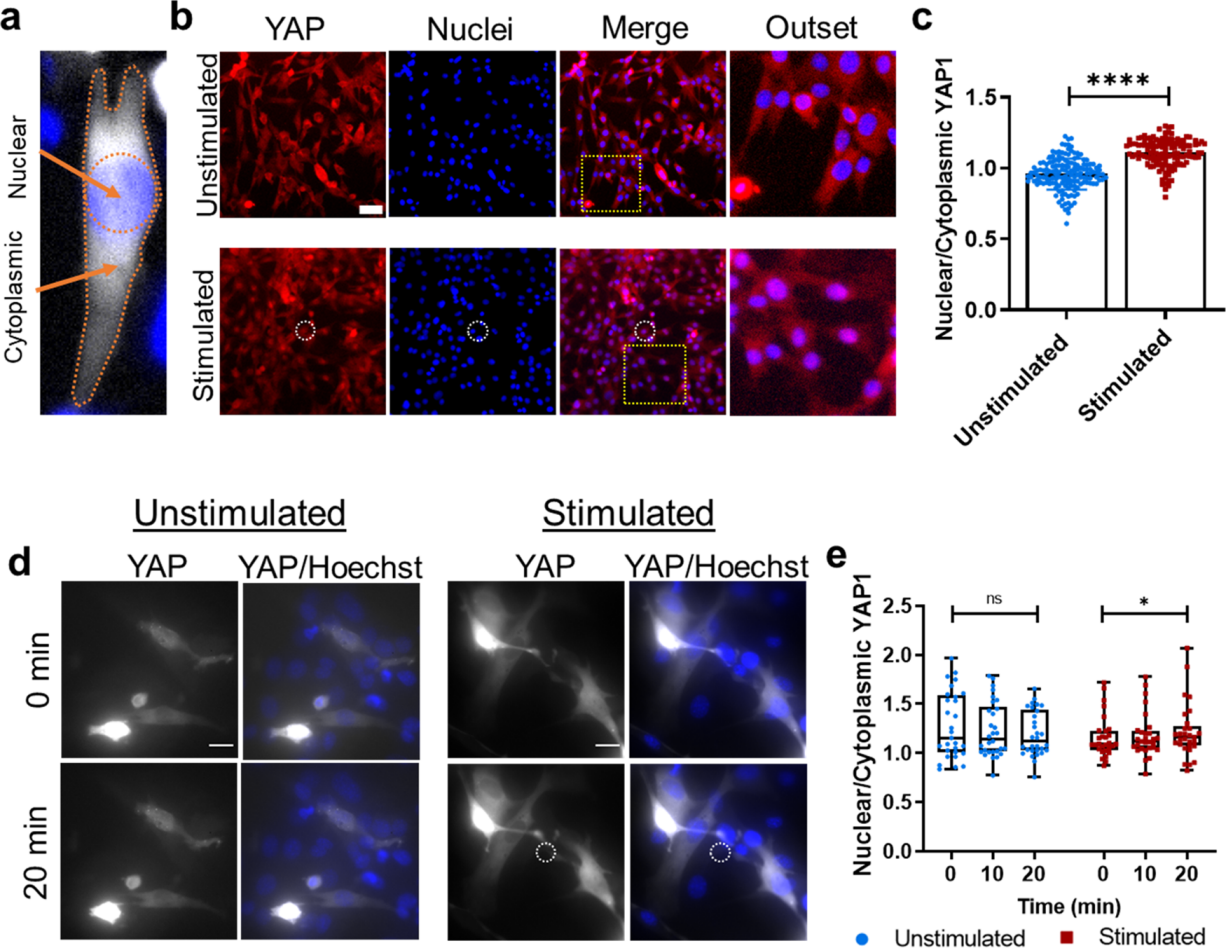
Effect of hydrogel actuation on YAP signaling. (a) Schematic demonstrating the method of nuclear and cytoplasmic fluorescence quantification in this manuscript. NucBlue (fixed cells) or Hoechst 33342 (live cells) was used to determine the area of the nucleus. Any cell fluorescence outside that region was characterized as cytoplasmic signal. (b) Representative images of C2C12 myoblasts stained for YAP1 (red) and counterstained for nuclei (blue) 18 h after receiving 20 min of NIR stimulation at 1 Hz (500 ms on time) and 33.1 μW/μm^2^ (bottom, white circle), or no stimulation (top). Scale bar: 20 μm. Outset image (yellow dotted square) shows overlapping red and blue staining in the nuclei of stimulated cells (bright purple nuclei, bottom). (c) Graph quantifying average nuclear-to-cytoplasmic YAP fluorescence ratio ± standard deviation. ****p < 0.0001 by Mann–Whitney test for at least 130 cells from *n* = 3 independently prepared experiments. (d) C2C12 cells transfected with EGFP-YAP1 were imaged during 20 min of no NIR stimulation (left) or NIR stimulation at 1 Hz (500 ms on time) and 33.1 μW/μm^2^ (white circle). Scale bar: 20 μm. (e) Box plot showing the range of nuclear-to-cytoplasmic YAP signal during stimulation (red) or no stimulation (blue). *p < 0.05 by mixed-effects model with Tukey’s multiple comparisons on 30 cells from *n* = 3 independently prepared hydrogel samples.

Having established morphological responses to mechanical stimulation, we next investigated myogenic signaling pathways that might be driven by oscillatory gel deformation such as YAP. Myoblasts stimulated on actuating hydrogels for 20 min with NIR, showed an ∼17% increased nuclear to cytoplasmic ratio of YAP fluorescence (Figure 4a) 18 h later compared to those on unstimulated gels (Figure 4b,c).

Next, to better understand the timescale on which YAP nuclear translocation occurs, C2C12s transfected with EGFP-YAP1 were imaged for 20 min under NIR stimulation. Unstimulated cells showed no significant change in nuclear to cytoplasmic YAP localization, but a slight increase in nuclear YAP was observed in NIR-stimulated cells (Figure 4d,e). However, when cells were fixed and stained for YAP1 18 h after stimulation, there was an even more significant increase in nuclear YAP compared to unstimulated cells (Figure 4b,c).

### Hydrogel Stimulation Affects Myoblast ERK Activation at Multiple Timepoints

**Figure 5:**
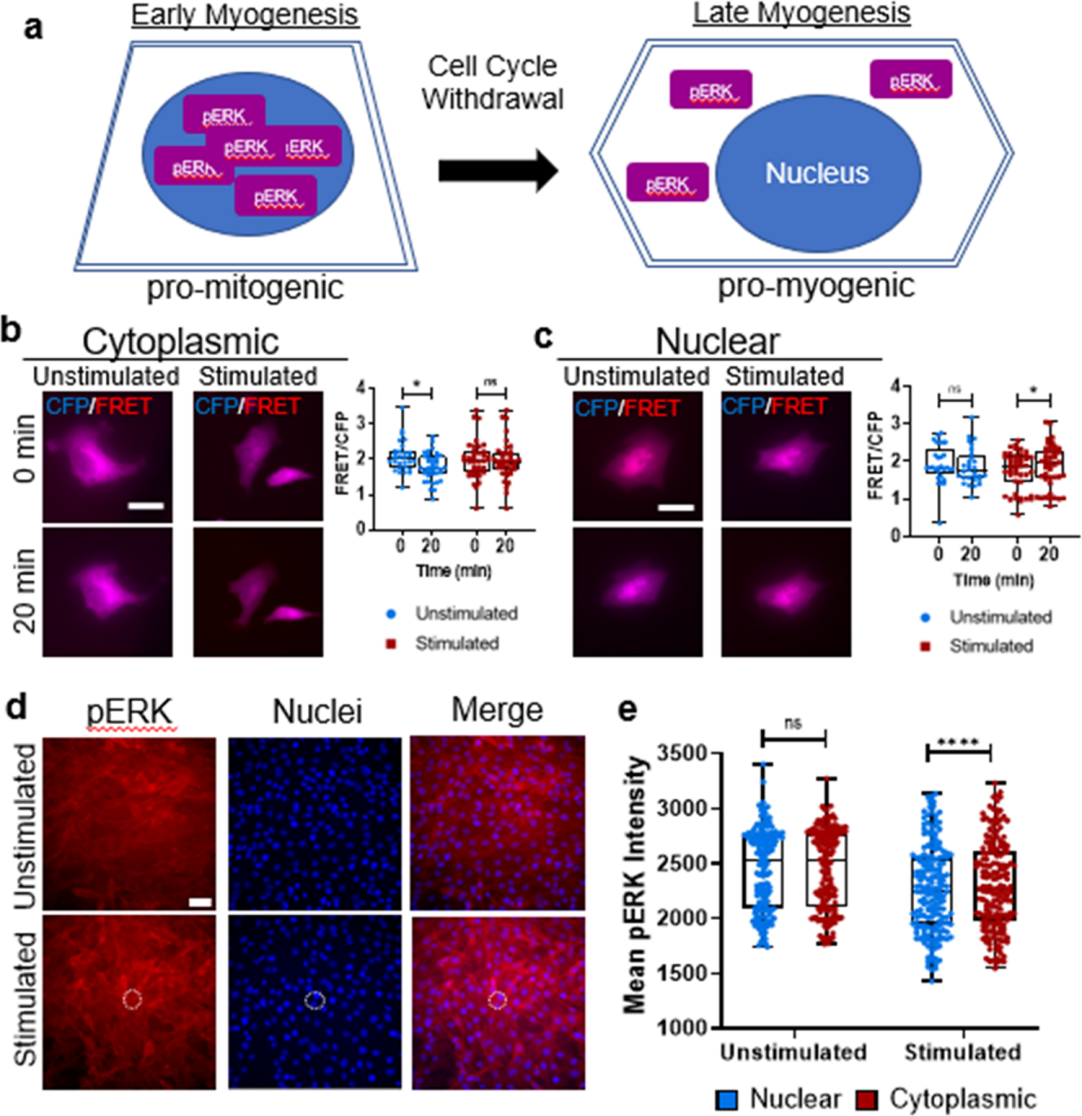
Effect of hydrogel actuation on ERK signaling. (a) Schematic drawing of ERK localization in early myogenesis (left) and late myogenesis (right). During differentiation, myoblasts withdraw from the cell cycle and mitogenic activity is reduced, with ERK activity moving from the nucleus to the cytoplasm. (b) (Left) Representative images of C2C12 myoblasts transfected with cytoplasmic EKAR reporter plasmid showing overlayed donor emission (blue) and sensitized FRET emission (red), either unstimulated or stimulated for 20 min with NIR light at 1 Hz (500 ms on time) and 33.1 μW/μm^2^ power density. Scale bar: 20 μm. (Right) Box plot shows the range of FRET ratio. *p < 0.05 by mixed-effects analysis with Sidak’s multiple comparisons for 40 cells from *n* = 3 independent experiments. (c, Left) Representative images of C2C12 myoblasts transfected with nuclear EKAR showing overlayed donor emission (blue) and sensitized FRET emission (red), either unstimulated or stimulated for 20 min with NIR light at 1 Hz (500 ms on time) and 33.1 μW/μm^2^ power density. Scale bar: 20 μm. (Right) Box plot shows the range of FRET ratio. *p < 0.05 by mixed-effects analysis with Sidak’s multiple comparisons for at least 30 cells from *n* = 3 independent experiments. (d) Representative images of C2C12 myoblasts stained by immunofluorescence for ERK phosphorylation (red) and counterstained for nuclei (blue) 18 h after 20 min of NIR stimulation at 1 Hz (500 ms on time) and 33.1 μW/μm^2^ power density (bottom, white circle), or surfaces that received no stimulation (top). Scale bar: 50 μm. (e) Box plot showing the range of pERK staining intensity ± standard deviation in nuclear and cytoplasmic regions of the cell. ****p < 0.0001 by a two-way analysis of variance with Sidak’s multiple comparisons for at least 165 cells from *n* = 3 independent experiments.

Because of the interaction of YAP signaling with ERK (or MAPK) in myoblasts,^32^ and because ERK signaling is an important component of early myogenesis^33,34^ (Figure 5a), we next investigated if mechanical stimulation of cells grown on actuating gels affected ERK activation. Cells were transfected with either nuclear or cytoplasmic EKAR, an engineered biosensor that, when bound to phosphorylated ERK, causes a conformational change that brings CFP and YFP molecules within FRET range, thereby providing a readout for ERK activation.^35^ After 20 min of NIR stimulation, cytoplasmic ERK activation was not affected, but nuclear ERK activation showed a slight but significant increase over the same period of stimulation (Figure 5b,c).

We then tested if phosphorylation would be upregulated on a longer timescale. Cells were fixed and stained for phosphorylated ERK (pERK) 18 h after NIR stimulation treatment. Mechanical stimulation significantly increased cytoplasmic pERK compared to nuclear pERK but suppressed pERK expression compared to unstimulated cells (Figure 5d,e).

### Actuating Hydrogels as an *In Vitro* Model of Exercise in Muscle

**Figure 6:**
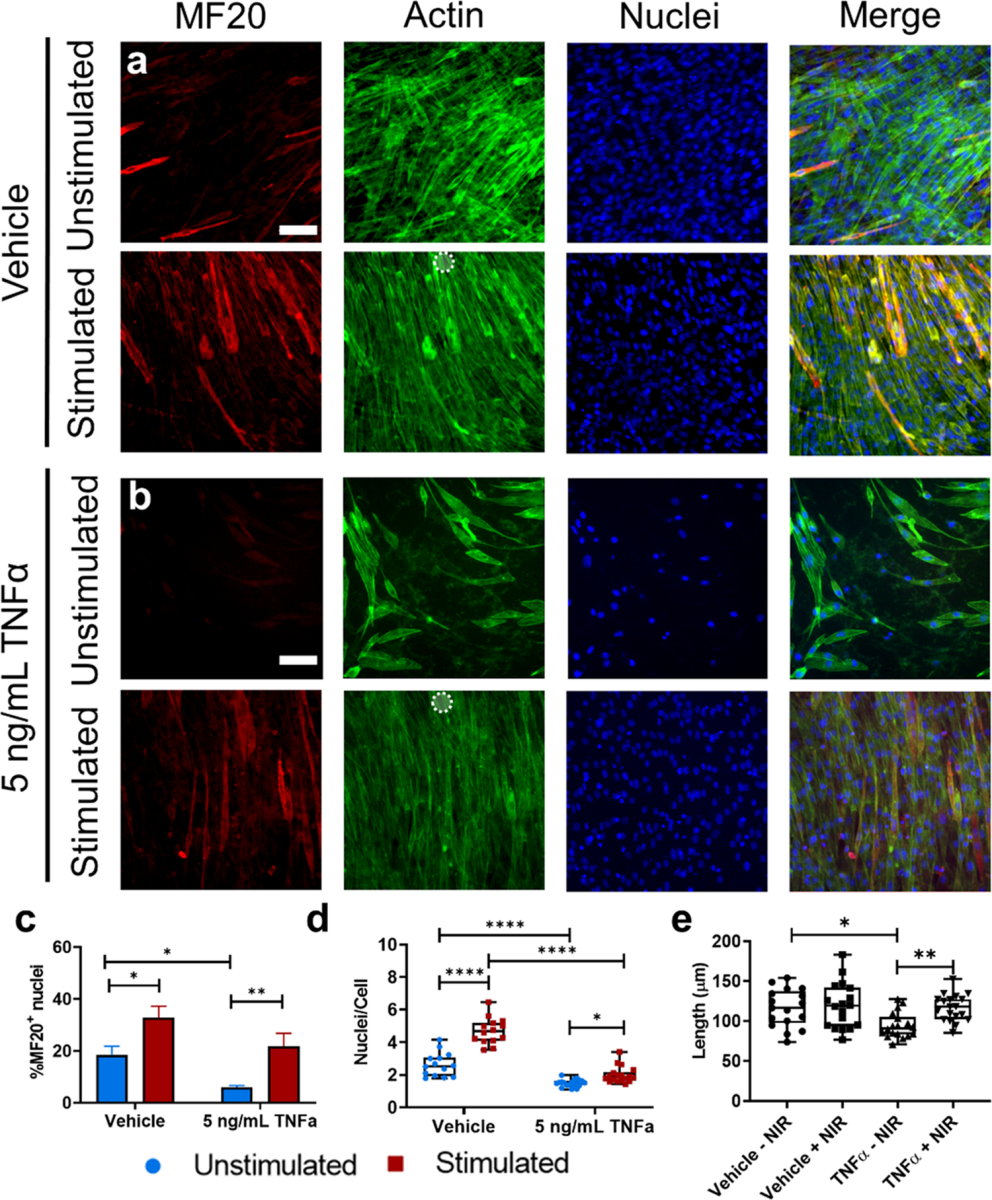
Effect of hydrogel substrate actuation on myogenesis. (a) Representative images of C2C12 myoblasts stained for MF20 sarcomeric myosin (red), actin (green), and nuclei (blue) after 5 days of NIR stimulation at 1 Hz (500 ms on time) and 33.1 μW/μm^2^ laser power (bottom) or cultured for 5 days with no stimulation (top), treated with a vehicle control of 1% BSA in 1× PBS. Scale bar: 50 μm (b) Representative images of C2C12 myoblasts stained for MF20 sarcomeric myosin (red), actin (green), and nuclei (blue) after 5 days of NIR stimulation at 1 Hz (500 ms on time) and 48 mW laser power (bottom) or cultured for 5 days with no stimulation (top), treated daily with 5 ng/mL mouse recombinant TNFα. Scale bar: 50 μm. (c) Bar graph showing mean ± standard error of the mean of positive MF20 myosin staining, quantified as % of total nuclei per ROI contained with myosin positive cells. **p < 0.01, *p < 0.05 by a two-way analysis of variance with Sidak’s multiple comparisons in at least 12 stimulated and unstimulated regions of interest taken from *n* = 3 independently conducted experiments. (d) Box plot quantifying fusion index as the average number of nuclei per cell in at least *n* = 12 stimulated and unstimulated regions of interest. ****p < 0.0001, *p < 0.05 by a two-way analysis of variance with Sidak’s multiple comparisons. (e) Box plot showing average cell length in at least *n* = 12 stimulated and unstimulated regions of interest. *p < 0.05, **p < 0.01 by Kruskal–Wallis test with Dunn’s multiple comparisons. All data were collected from 3 independent experiments.

Finally, we quantified later markers of myogenesis—multinucleation and myosin expression—and investigated the ability of strain-producing hydrogels to rescue reduced myofiber formation due to chronic inflammation. C2C12s were cultured on actuating hydrogels, treated with either TNFα or equal volume of drug vehicle, then cultured with or without NIR stimulation every other day for 5 days (Figure S12). “Uninjured” cells (cells with vehicle treatment) showed an increase in both myosin expression and fusion as a result of mechanical stimulation (Figure 6a,c,d). Daily treatment with TNFα, a model of chronic inflammation, significantly decreased both myosin expression and cell fusion compared to the control as expected. However, NIR mechanical stimulation rescued these phenotypes, though the increase in fusion was not statistically significant (Figure 6b–d).

## Discussion

We have previously reported the ability of OMAs to mechanically stimulate single cells on 2D substrates,^27,28^ and, in this work, we sought to expand these capabilities by incorporating actuating nanoparticle elements into a hydrogel-based cell culture substrate, GelMA (Figure 1a). OMAs were functionalized with methacrylate groups in order to physically link actuating nanoparticle elements into the hydrogel network (Figure S2a–c). Because this physical linkage was crucial to force transduction within the gel, methacrylate addition to OMAs was validated by fluorescence of fluorescein-o-methacrylate (Figure S2d–f). Notably, aggregates of MA-OMAs were observed at high magnification, likely due to methacrylate-mediated OMA–OMA crosslinking, further confirming functional methacrylate groups were present on the OMA surface. SEM also confirmed OMA incorporation, revealing a textured gel surface with spherical structures distinctly different from other reported electron microscopy images of GelMA hydrogels (Figure 1c).^37^

Because GelMA is a degradable polymer, we investigated the degradation of actuating hydrogel constructs in storage conditions (4 °C) and experimental conditions (37 °C) (Figure S3). Less degradation was observed in higher OMA-content gels in both conditions. This is further, though indirect, evidence of methacrylate modification of the OMAs and their incorporation into the gel. Gels with more OMAs, and therefore more methacrylate groups, may form more crosslinks in the gel formation process, contributing to this stability. However, this experiment did not include any proteases that would be found in a biological environment, where GelMA constructs are reported to completely degrade in 2–4 weeks.^38^ Such degradation could compromise the force transmission of the OMAs to the gel matrix, though this timescale is beyond that investigated in this work.

Importantly, OMA thermoresponsivity was found to be maintained when embedded in GelMA (Figure S1c,d). This property is crucial to the functionality of the OMA, as it is the photothermal heating of the AuNR core of the nanoparticle in response to NIR illumination that drives the mechanical actuation. This property also allowed us to study changes in the mechanical properties of the actuating hydrogel between its relaxed and contracted states.

GelMA hydrogels have been well characterized, and the Young’s modulus of 10% w/v GelMA was reported as 13 kPa using AFM,^39^ and even higher by uniaxial tensile testing.^10^ This concentration of GelMA is often used for the study of myoblasts and myogenic stem cell differentiation on hydrogels.^40−42^ We investigated if the addition of OMAs would alter the mechanical properties of the gel when the temperature exceeded the transition temperature of the OMAs, that is, when the actuating elements collapsed. Because *G*′ did not change significantly as a function of OMA collapse, observed responses in subsequent experiments can be attributed to gel deformation, not changes in mechanical properties (Figures 1d and S4). Interestingly, the temperature-dependent *G*′ response of actuating gels was more variable than that of GelMA without OMAs.

Though these gels can be actuated by bulk heating, we mainly characterized NIR light-driven photoresponsivity in this work. This property is desirable compared to bulk thermoresponsiveness for several reasons. Primarily, optical triggering provides precisely localized stimulation and rapid responsivity.^26^ Our previous work showed that OMAs display the greatest reported temporal response due to the unique geometry encapsulating the AuNR and heating “from within”, which was orders of magnitude faster than external “bulk” heating.^30^ Moreover, slow bulk thermal diffusion rates throughout a hydrogel limit spatial control.^43^

Interestingly, the magnitude of *Z* deformation was greater than that observed in the *XY* plane (Figure 2d,f). This may be due to a number of factors. One is the point spread function of the laser which allows for greater excitation in the *Z*direction compared to the *XY* plane. Another is that the source of illumination coming from below the sample. The third contribution is likely the gel geometry which is more pinned in the *XY* plane in contrast to the *Z* direction. The data also suggests the high *Z* deformation near the NIR illumination causes a point source deformation that stretches the surface of the gel to accommodate the volume change. This creates a gradient of deformation radiating outward from the NIR laser illumination site such as that we observe, causing illumination of a relatively small region of interest to result in deformations tens to hundreds of microns away. This unique 3D deformation profile in the hydrogel, which was found to be highly tunable by changing both OMA content and stimulation intensity, allows the application of strain to cells farther from the NIR stimulus than OMAs alone, increasing throughput compared to our previous work (Figure 2e,g–i).^28^

Notably, when analyzing the strain fields within the actuating hydrogel, we assumed the gel to be nearly incompressible and linearly elastic (Poisson’s ratio *ν* = 0.495), as GelMA has been shown to have an almost purely elastic strain response (<0.5% viscoelastic strain when held under constant stress for 5 min^44^) (Figure 2e,g,i). The magnitude of the stress calculation is likely an underestimate, given our measured elastic modulus was lower than reported elsewhere.

Still, by applying deformation to substrates on this scale—within tens of microns with lower NIR laser intensity stimulation and greater than 100 μm at higher NIR laser intensities—the present work also represents an improvement in spatial resolution compared to CSBs. These instruments are beneficial in many applications by applying strain to many cells at once, but the entire substrate must be actuated. This limits spatial control of mechanical stimulation in CSBs. The novel, OMA-containing actuating hydrogel offers a highly tunable range of strained areas on a single substrate, precisely controlled by the location and intensity of an input NIR stimulus.

The observed deformations in our material are comparable to other photothermally actuated hydrogel systems, such as the HAIRS system, which shows in-plane displacements of 2–8 μm, dependent on AuNR content.^26^ However, that work transfers the deformation to micropillars, which provide discreet “islands” for cell adhesion, while the actuating hydrogel in the present work supports cell adhesion across the entire material surface, providing a more continuous and physiologically relevant force application to cells.

Another advantage of photothermal triggering of mechanically active hydrogels over bulk heating above the LCST of pNIPMAm (>42 °C) is the ability to perform cell culture experiments without the heating demonstrated to cause non-physiological signaling and even cell death.^48,49^ To validate the suitability of our actuating hydrogel for cell culture, we quantified thermal accumulation in our system with rhodamine, a temperature-sensitive fluorescent molecule with a known reduction in quantum yield of −0.93% per °C increase.^50^ Minimal heat accumulation was observed, consistent with thermodynamic modeling of OMAs showing little to no heating on the surface of the nanoparticle (Figure S9a–d).^27^

We did not expect the heating observed under the experimental conditions in this work (1 Hz, 50% duty cycle, 33.1 μW/μm^2^) would be confounding to biological outcomes, given that experiments were performed at room temperature. To verify this, we conducted a cell viability assay by incubating cells in a solution of DAPI. DAPI is impermeant to live cells and is known to greatly increase in fluorescence when bound to DNA within the cell. Heating above 37 °C has been demonstrated to increase cell membrane permeability within 20 min.^51^ Therefore, the lack of intracellular DAPI signal suggests the experimental conditions in this work do not induce cytotoxicity (Figure S9e).

To further ensure the actuating hydrogel substrate was suitable for cell culture, laminin was incorporated by its previously demonstrated binding to the NH_2_ terminus of collagen molecules.^52^ Laminin and collagen are both important components of the muscle ECM,^45^ and coating culture surfaces with gelatin or laminin is common in muscle cell culture.^46,47^ In this work, the addition of laminin to the gelatin substrate was hypothesized to improve myoblast attachment by creating a more biomimetic environment (Figure S8).

Notably, fibronectin is also an important ECM protein in muscle tissue,^45^ and previous work from our group and others’ cultures myoblasts on surfaces coated with the fibronectin adhesion domain, RGD.^28,53^ Integrin receptor expression in muscle cells is dynamic throughout the process of myogenesis, with higher expression of laminin-binding integrins as cells mature.^54,55^ Future work with this material could study how fibronectin or RGD, exclusively or in addition to laminin, may augment the response of myoblasts to actuating hydrogels at earlier timepoints.

However, C2C12 cells not only attached to the actuating hydrogel substrates but also responded to the NIR-driven mechanical stimulation. Cells elongated in response to stimulation on actuating gels, consistent with our previous work in fibroblasts^27^ and myoblasts.^28^ The observed responses also reflect those in the literature culturing myoblasts on CSBs.^13,14^ Cells stimulated on AuNR gels did not behave differently than those on unstimulated gels (Figure S10c,d), isolating the observed response on OMA-containing gels to mechanical stimulation from the hydrogel and not photothermal heating.

Given the cells responded with rapid changes in morphology, we investigated what signaling pathways might be activated by the mechanical actuation of the hydrogel. YAP, a mechanotransductive transcription factor,^56,57^ can enter the nucleus following mechanical perturbation. These forces are thought to open nuclear pores to allow transport of YAP from the cytoplasm.^25^ NIR stimulation does form a gradient of deformation across tens to hundreds of microns of the gel surface causing dynamic mechanical stress that would deform the cell. Live YAP reporter results suggest cells may begin responding to mechanical stimuli after as little as 20 min of perturbation (Figure 4d,e).

Fixing and staining for YAP after stimulation showed even more defined nuclear translocation. This may be due to differences in staining efficiency or signal strength between the expressed fluorophore and the fluorescent antibody. Indeed, GelMA substrates were made with Eosin Y, which may have contributed a higher background fluorescence to the EGFP. However, it is also possible that additional mechanisms may continue upregulating this mechanosensitive pathway after the stimulus has subsided. The extended timescale of YAP accumulation in cells is outside of the scope of the current work but would be an interesting area of future study.

YAP signaling has also been linked to ERK activation in myoblasts. Because ERK signaling is important early in myogenesis, this suggests a pathway for mechanosensitivity of myogenesis. Michailovici et al. showed that pERK localization within myoblasts and myocytes changes over time during myogenesis, with higher nuclear expression early on, then moving to the cytoplasm in later differentiation.^58^ Other work has indicated that ERK activation is decreased overall in more mature myocytes.^59^ Therefore, we again used transfected fluorescent probes and immunostaining to visualize the amount and location of ERK phosphorylation (Figure 5). Though the EKAR probe results alone did not strongly suggest mechanically mediated ERK activation during actuation, these data in combination with immunostaining suggested actuating hydrogels drive myogenesis by increasing nuclear ERK activation early on, then increasing cytoplasmic ERK activation some hours later to promote differentiation (Figure 5a).

Later stages of myogenic differentiation are marked by the fusion of myocytes into multinucleated myotubes and myofibers which express myosin, a protein responsible for the force production of muscle. Having established that actuating hydrogels modulate early myogenic signaling pathways, we next investigated whether mechanical stimulation captures some of the features of mechanically enhanced myogenesis observed *in vitro*, *in vivo*, and clinically,^13,14,28^ using our actuating hydrogel as the source of mechanical strain in lieu of a CSB or exercise. Indeed, exercise is shown to alleviate inflammatory damage to muscle,^36^ such as the decreased myogenesis observed under prolonged inflammation,^60^ improving muscle healing and force production after injury.^61,62^

The advantages of studying cells in a mechanically active environment extend beyond exercise science to modeling pathologies and therapies *in vitro*. Impaired force transmission from the extracellular space causes pathologic phenotypes in the muscle,^63^ and *in vivo* and clinical work has repeatedly demonstrated that the repeated application of forces to the muscle is therapeutic in cases of musculoskeletal injury and disease.^61,64^ Research into the mechanism of these observed effects has implicated the reduction of inflammatory damage in muscle proteins following injury, including that caused by the pro-inflammatory tumor necrosis factor α (TNFα).^36^

In our actuating gel system, mechanical stimulation seemed to recover myogenic phenotypes in a TNFα-treated chronic inflammation model. Notably, fields of view were selected randomly, and many elongated myotube structures extend outside the field of view of the collected images (Figure 6). Thus, the reported fusion quantification is likely an underestimate of actual number of nuclei per cell.

Though our model does not accurately capture the biophysical and biomechanical intricacies of repetitive muscle contraction in the body, our results align with previous work showing a protective effect of exercise in preventing TNFα-dependent suppression of myogenesis.^36^ In this way, we demonstrate actuating hydrogels as a tool for inducing myotube formation by accelerating myogenesis *in vitro*, and our results further suggest the mechanical activity of this material may be able to model *in vitro* some aspects of the mechanical effects of exercise in muscle biology and healing.

### Future Directions

Recently, one group 3D-printed GelMA fibers containing myoblasts and further promoted cell alignment through the application of an electric field during the printing process, resulting in increased myogenesis.^40^ This combination of printed, aligned, cell-laden structures with additional stimulation is highly representative of next steps in developing the techniques described in this work. As preliminary validation of this possibility, we 3D-printed actuating hydrogels containing 10% w/v GelMA and 20% OMAs, and observed actuation of the gel under NIR illumination (Figure S11c,d and Video S2). As an additional demonstration of future directions, we incorporated OMAs into other hydrogel materials, both biological (collagen 1) and synthetic (PEG). We found both gel types actuated with NIR illumination (Figure S11a,b and Videos S3 and S4).

The present work is limited to the characterization and demonstration of a mechanically active hydrogel system with uniformly dispersed actuating elements. However, these alternate techniques for forming the gel could also expand the capabilities of this system. For example, 3D printing could be used to vary the concentration of OMAs spatially within the gel. Patterning areas of higher and lower concentration could allow even further spatial control over strain application to cells grown on the hydrogel. Traditional techniques to create gradients of nanoparticles within the gel, such as the application of magnetic fields, would not work on the gold-polymer composite OMAs. As such, 3D printing with “inks” of varying OMA concentrations in GelMA could allow for more complex strain responses within the gel.

These demonstrations show OMAs are modular components that can confer optomechanical responsivity to multiple different hydrogels. These results also demonstrate the broad responsivity of this actuating GelMA system, and the broad parameter space of OMA contents and laser powers can precisely tune this material for multiple applications.

Soft materials such as hydrogels have broad applications in the field of biomaterials beyond the biological hypotheses in this work. Remotely controlled hydrogels could be potentially useful as actuating elements in soft robotics, gates in microfluidic systems, or engineering tissue constructs in dynamic environments.

## Conclusions

In this work, we present a novel, light-actuated hydrogel nanocomposite as a tool to study the role of extracellular forces on muscle cells *in vitro*. The responsivity of these gels is highly tunable by both NIR intensity and OMA concentration, providing cyclic deformation on the order of microns to tens of microns. We also demonstrated the ability of these actuating materials to affect muscle cell biology, exhibiting pro-myogenic phenotypes at multiple timescales (minutes, hours, and days), comparable to more complex actuation systems such as CSBs.^13,65^ Importantly, the actuation provided by these materials is shown to help recover the myogenic phenotype in cells treated with TNFα, mimicking the results of exercise in chronic inflammation models. Because of this, we propose the actuating material system described herein could be applied to study the beneficial effects of exercise in muscle physiology, pathology, and injury *in vitro*. Additionally, we demonstrate that this OMA hydrogel system can be applied to other hydrogel substrates besides GelMA, which could expand the applications of this method in studying cell biology as well as other fields within biomaterials engineering.

## Materials and Methods

### OMA Synthesis and Characterization

AuNRs were synthesized as described previously.^27,28^ Briefly, a gold nanorod seed solution was prepared by combining 5 mL of 0.5 mM HAuCl_4_ (Acros Organics, Thermo Fisher Scientific) and 5 mL of 0.2 M CTAB (TCI). 600 μL of 0.01 M NaBH_4_ (Sigma-Aldrich) was diluted to 1 mL and added quickly to the gold solution and stirred vigorously at 1200 rpm for 2 min, then left undisturbed at room temperature for 30 min.

3.6 g of CTAB and 0.4936 g of sodium oleate (TCI) were dissolved in 100 mL of water at 60 °C and stirred at 1000 rpm. After cooling to room temperature, 8.5 mL of 4 mM AgNO_3_ (Sigma-Aldrich) was added and was left undisturbed for 15 min. Subsequently, 4 mL of 25 mM HAuCl_4_ was diluted to 100 mL and added to the CTAB solution and stirred for 90 min at 700 rpm. 600 μL of HCl was added and stirred for 15 min, then 600 μL of 64 mM ascorbic acid was stirred in vigorously for 30 s. 80 μL of seed solution was added, stirred for 30 s, and then left undisturbed for at least 12 h at 30 °C. AuNRs were centrifuged at 5000 rpm for 60 min, then resuspended in 90 mL of water.

To facilitate OMA polymerization, AuNRs were modified with a thiolated vinyl terminal by adding 20 mg of *N*,*N*′-bis(acryloyl)cystamine (Alfa Aesar) dissolved in 10 mL of ethanol to the 90 mL AuNR solution and stirred vigorously for 24 h. The resultant solution was concentrated 5×. The pNIPMAm shell was created by heating 0.1 g of *N*-isopropyl methacrylamide (Sigma-Aldrich) and 0.01 g of *N*,*N*′-methylenebisacrylamide in 15 mL of water to 70 °C in a three-neck flask under continuous N_2_ flow and stirring. 1 mL of AuNR solution was added, and polymerization was initiated with 80 μL of 0.1 M 2,2′azobis(2-methylpropionamidine) (Sigma-Aldrich). After 2 h, 20 μL of propargyl methacrylate in 1 mL of ethanol was added, and the mixture was maintained at 70 °C for 1 h. OMAs were washed twice by centrifugation at 5000 rpm for 60 min and resuspended to a final concentration of 14.5 nM. OMAs size was validated by TEM (Hitachi) and dynamic light scattering (DLS, NanoPlus Zeta/Particle Analyzer, Particulate Systems). For the synthesis of control nanoparticles, the same protocol was followed, without the addition of AuNRs.

An azide-modified methacrylate was synthesized by combining 2 mg of 2-aminoethyl methacrylate hydrochloride (Sigma-Aldrich) with 2 mg of NHS-Azide (Thermo Fisher) in 500 μL of DMSO with 4 μL of diisopropylethylamine (DIPEA) to provide basicity. The NHS-amine reaction was allowed to proceed overnight. The mixture was filtered through a 0.2 μm centrifugal filter, and the filtrate was purified by reversed-phase HPLC (Alltima C18 5u, Alltech, 4.6 × 250 mm^2^, 1.0 mL/min flow rate; solvent A: 0.1 M TEAA in water, solvent B: acetonitrile; starting condition: 90% A + 10% B, 1% per min gradient B for 15 min). The product-containing fraction was lyophilized to obtain 8.9 mg of azido-methacrylate (42 μmol, 70%), and was subsequently characterized by NMR: ^1^H NMR (400 MHz, DMSO-*d*_6_) δ 8.28 (t, *J* = 5.6 Hz, 1H), 6.06 (dq, *J* = 1.7, 1.0 Hz, 1H), 5.69 (dq, *J* = 1.7, 1.6 Hz, 1H), 4.11 (t, *J* = 5.6 Hz, 2H), 3.83 (s, 2H), 3.39 (q, 5.6 Hz, 2H), 1.88 (dd, *J* = 1.6, 1.0 Hz, 3H). Finally, an electrospray ionization (ESI) mass spectrum was obtained to validate the product, which showed a peak at 235.08011 *m*/*z* corresponding to the expected molecular mass of 235.0802.

OMAs were modified with methacrylate groups by copper-catalyzed azide-alkyne cycloaddition click reaction: 50 μL of 121 mM azide-methacrylate was added to the alkyne-OMA solution, along with 20 μL of THPTA (Lumiprobe), 10 μL of CuSO_4_ (Mallinckrodt), and 30 μL of sodium ascorbate (Sigma-Aldrich). The solution was incubated overnight on an orbital shaker, then OMAs were washed by centrifugation (5000 rpm for 10 min) three times with 1× PBS, and resuspended to a final concentration of 14.5 nM in appropriate buffer (1× PBS or potassium phosphate buffer (100 mM, pH 6.4)).

To validate incorporation of methacrylate groups onto OMA surfaces, 20 μL of methacrylated or nonmethacrylated OMAs was combined with 14.5 μM fluorescein-O-methacrylate (Sigma-Aldrich), 1 μL of 0.01 mM Eosin Y (Santa Cruz Biotechnology), 0.37 μL of 0.375% v/v 1-vinyl-2-pyrrolidione (NVP, Sigma-Aldrich), and 1.5 μL of 1.5% v/v triethanolamine (TEOA, Sigma-Aldrich) and incubated under a white light source for 10 min to crosslink fluorescein to the particles. Particle-containing solutions were imaged using a Nikon Eclipse Ti epifluorescence microscope (Nikon) at 100× using FITC excitation/emission.

### Hydrogel Synthesis

A hydrogel crosslinking solution was prepared with 2.5 μL of 0.01 mM Eosin Y, 0.938 μL of 0.375% v/v NVP, and 3.75 μL of 1.5% v/v TEOA in 142.8 μL of 1× PBS with 1% v/v penicillin–streptomycin (Corning). 25 mg (10% w/v) of gelatin methacrylate (GelMA, Allevi or Cellink) was added to the crosslinking solution, and incubated at 60 °C until dissolved. Next, a solution of 14.7 nM OMAs was added to achieve a final concentration of 10, 15, or 20% v/v in the gel. Finally, mouse laminin (Corning) was added to a concentration of 1 μg/cm^2^.

Two glass surfaces (#2, 25 mm round slides, VWR) were prepared to form flat hydrogel surfaces. One slide was cleaned in piranha solution (sulfuric acid and hydrogen peroxide) to expose silane groups, and then was incubated for 20 min in 0.4% v/v 3-(trimethoxysilyl)propyl methacrylate (Sigma) in acetone, as described elsewhere.^66^ A second cover glass was prepared by submersion in a 1% solution of SurfaSil (Thermo) in acetone, rinsed for 5–10 s in acetone, rinsed briefly in methanol, and then dried for 60 min to cure the surface.

Hydrogel surfaces for cell culture were prepared by adding 50 μL of GelMA solution to the methacrylate-modified glass slide and sandwiching with the siliconized slide using a spacer of Parafilm (Bemis) to create gels of approximately 100 μm thickness. Hydrogel sandwiches were allowed to rest at 4 °C for 10 min and then photocrosslinked using a fiber optic white light source for 10 min. After 20 min of incubation at 4 °C, the siliconized slide was removed. Gels were imaged by scanning electron microscopy.

In the case of microscopic characterization of deformation, the siliconized slide was replaced with a glass slide coated with poly-l-lysine (Thermo) for 30 min, then coated with 0.5 μm Tetraspeck beads (Thermo) for 1 h as described elsewhere.^67^ This created a single layer of fluorescent beads on the top surface of the gel upon removal of the poly-l-lysine slide.

Additional OMA hydrogels were prepared using collagen (rat tail collagen 1, Corning) by combining 40 μL of stock solution with 10 μL of 14.7 nM OMA solution in 1× PBS, and then polymerized in a basic environment of ammonia gas for 2 min.

To create PEG hydrogels, two separate precursor solutions were made by dissolving 6 mg of tetraPEG-NH2 (MW ∼10 kDa, Laysan Bio, Inc.) and 6 mg of tetraPEG-NHS (MW ∼10 kDa, NOF America Corp.) into separate 50 μL aliquots of 14.7 nM OMA 20% v/v in potassium phosphate buffer (100 mM, pH 6.4) and kept on ice. Mouse laminin and cRGDfk-NH2 were dissolved in buffer solution to final concentrations of 100 μg/mL and 2 mM, respectively, then 20 μL of this solution was mixed into the tetraPEG-NH_2_ solution. Finally, the solutions were combined and mixed by vortexing for 10 s. 45 μL of the mixed solution was transferred onto a smooth parafilm surface and then sandwiched between glass and parafilm surface to form the PEG hydrogel.

### NIR Stimulation

In this work, we used a Nikon Eclipse TI inverted microscope (Nikon) equipped with a glavo illuminator (Rapp Optoelectric) that generated an ∼40 μm laser spot (λ = 785 nm) through a 20× air objective (NA = 0.50). This system allowed for simultaneous NIR illumination and fluorescence imaging. Stimulation was provided to surfaces as a step function of laser intensity at 1 Hz with 50% duty cycle, unless otherwise noted.

### Material Characterization

To quantify local heating at the surface of the gel, gels were submerged in a solution of 100 nM tetramethylrhodamine (Thermo Fisher), and gel surfaces were imaged using fluorescence microscopy with TRITC excitation/emission specifications. Gels were excited with the 785 nm NIR laser at 1 Hz with duty cycles of 10, 50, and 90%, and laser powers of 25, 50, 75, and 100% (corresponding to 4.8, 15.8, 26.9, and 33.1 μW/μm^2^, respectively), and average intensity of TMRM signal was compared in conditions of NIR on and NIR off.

To examine any changes in material stiffness due to OMA collapse, rheological tests were carried out on an AR2000ex rheometer equipped with a temperature controller. Experiments were performed by sandwiching hydrogel samples between the temperature-controlling stage and a 25 mm stainless steel parallel plate. To compensate for the temperature-driven shrinkage of the material, all of the tests were performed under a constant normal force mode (1 ± 0.1 N) at a fixed oscillation strain of 1%. Temperature sweep tests were carried out at a fixed frequency of 6.28 rad/s within the temperature range of 25–50 °C.

Finally, hydrogel constructs with varying concentrations of OMAs were incubated at 4 and 37 °C for 3 weeks in DMEM with 1% penicillin/streptomycin. Gel weights were measured weekly, and the media was changed to prevent bacterial contamination.

### 3D Printing

OMA-GelMA solutions containing 10% w/v GelMA with 20% v/v OMAs were printed using a BioX 3D bioprinter with a temperature-controlled pneumatic printhead fitted with sterile 27G precision nozzle tips (Cellink). GelMA, crosslinkers, and OMAs were mixed as described above at 37 °C, transferred to a BioX print cartridge, and cooled at 4 °C for 5 min. The ink was then heated to 26 °C for 20 min in a temperature-controlled pneumatic printhead in the BioX printer. The print bed was cooled to 4 °C. Constructs were printed at 30 kPa pressure and 15 mm/s in a square lattice shape (10 mm × 10 mm) at 40% infill density with 200 μm feature size and two-layer construct height. Gels were crosslinked with white light at 4 °C for 5 min. All constructs were kept in PBS with 1% penicillin–streptomycin (Corning) and kept in cell culture incubators (37 °C) prior to imaging.

### Actuation Characterization

We validated gel responsivity by heating hydrogel droplets (formed by applying hydrogel precursor solution to a glass slide and not flattening the resulting droplet) to 50 °C on a heated stir plate and photographed with an iPhone 8 camera (Apple). The apparent area of the gel was determined by taking two perpendicular diameter measurements of the gel and calculating the area of an ellipse. The apparent gel area was measured at room temperature (25 °C) and 50 °C.

To measure gel deformation, hydrogels prepared with a fluorescent bead layer, as described above, were imaged on a Nikon Eclipse Ti Epifluorescence microscope with a 40×, 0.95 NA objective and TRITC excitation–emission wavelengths. Z-stack 3D images were obtained using a slice size of 0.5 μm, acquiring enough slices to capture the entire point spread function of the microbead fluorescence (>20 μm total stack height). First, an image with no NIR illumination was obtained, then additional z-stacks were imaged at varying NIR power intensities. Images were preprocessed in ImageJ using a 50-pixel rolling-ball background subtraction.

To resolve volumetric gel deformations, we utilized the recently developed augmented Lagrangian digital volume correlation (ALDVC) algorithm to track the volumetric deformation fields of fluorescent beads attached to gel surfaces, comparing volumetric images features before and after applied NIR stimulus to computationally infer displacement and strain fields within the gel.^31,68^ The size of each local 3D window was set at 40 × 40 × 20 voxels (16 × 16 × 10 μm^3^), with each window 50% overlapped with its neighboring windows and a DVC window spacing of 20 × 20 × 10 voxels (8 × 8 × 5 μm^3^). After solving the full-field deformations, 3D displacement fields were extracted at the plane of the fluorescent beads (i.e., the top surface of the gel) and plotted in 3D. The strain was determined from the deformation gradient within the gel, and von Mises stress was calculated assuming linear elasticity using our measured *G*′ of 967 Pa.

### Cell Culture

C2C12 myoblasts were maintained in culture at 37 °C with 5% CO_2_ concentration in growth media (DMEM (Gibco) supplemented with 10% fetal bovine serum (Corning) and 1% penicillin–streptomycin (Corning)), and passaged no more than 15 times before use in experiments. Glass slides with hydrogels were fitted into metal imaging chambers and cells were plated at a density of 60,000 cells/chamber in growth media and allowed to attach overnight before experiments were conducted. To validate cell adhesion to actuating hydrogels, gels were formed with and without laminin, and adhered cells were visualized with 10 μM CellTracker CMTMR (Invitrogen). The number of attached cells and cell spreading area were quantified.

For experiments longer than 1 day, cell media was changed daily, using growth media until cells reached ∼80% confluency, at which point media was changed to DMEM supplemented with 2% donor horse serum (Corning) and 1% penicillin–streptomycin.

### NIR Stimulation

To facilitate observing live-cell responses to hydrogel actuation, C2C12 myoblasts were transfected with mCherry LifeAct, cytoplasmic EKAR (cerulean-venus) (gifted to Addgene by Karel Svoboda, Addgene Plasmid #18679^35^) according to the protocol established by Mercer et al.^69^ Briefly, for each surface, empirically determined amounts of plasmid (1000 ng of mCherry LifeAct, or 1500 ng of EKAR plasmid) were mixed by pipetting with 100 μL of Opti-MEM (Gibco) and 2 μL of Lipofectamine 2000 (Invitrogen) per μg of DNA. After incubation at room temperature for 25 min, plasmid solutions were added to surfaces concurrent with cell plating and incubated overnight before imaging. Cells were kept at room temperature for the duration of NIR stimulation and then returned to 37 °C. Stimulation was performed at 1 Hz with 50% duty cycle (500 ms on-time) at 33.1 μW/μm^2^ for 20 min in each region of interest. Cells imaged for live fluorescence were counterstained with Hoechst 33342 (Thermo Fisher, Waltham, MA). To investigate cytotoxicity, DAPI was added to the cell media to a final concentration of 1 μg/mL before NIR stimulation. DAPI fluorescence was then imaged to determine cellular uptake as a marker of cell damage.

In some cases, cells were not transfected but were still stimulated for 20 min. To provide a fiducial marker for repeated imaging in the NIR-stimulated regions, a pattern was scratched onto the back of cell-seeded GelMA slides with a diamond scribe (Figure S12). The cells were then incubator for 18–24 h, and subsequently fixed for 20 min in 4% formaldehyde in 1× PBS and stained for phosphorylated ERK as well as YAP localization, following the protocol described below.

In other cases, cells were not transfected but were still stimulated for 20 min. To provide a marker for repeated NIR stimulation, subsequently, a pattern was scratched onto the back of cell-seeded GelMA slides with a diamond scribe (Figure S12). Surfaces were exposed to NIR stimulation of 1 Hz (50% duty cycle, 33.1 μW/μm^2^) for 20 min every other day at the “X” marked locations for 5 days (Figure S12).

Mouse recombinant TNFα (100 μg/mL with 1% BSA in 1× PBS, Corning) was diluted in cell media to a final concentration of 5 ng/mL, and cells were treated daily beginning after the first stimulation. An equal volume of 1% BSA in 1× PBS was added to cell media as a vehicle control. Treatment was continued daily for the 5-day duration of the experiments, with fresh media added after each NIR stimulation. After 5 days, the cells were washed three times in 1× PBS and fixed in 4% formaldehyde (Sigma-Aldrich) in PBS at room temperature for 20 min.

### Immunocytochemistry

Triton X-100 (0.1% in 1× PBS) (Sigma) was used to permeabilize cells, then blocked for 30 min with 1% BSA (Roche, Switzerland), 22.5 mg/mL glycine, and 0.1% Tween 20 (Sigma) in PBS. The following primary antibodies were used:

To visualize sarcomeric myosin expression, anti-MF20 (contributed to Developmental Studies Hybridoma Bank by Fischman, D.A.^70^) was diluted 1:2 in 1% BSA in PBS with 0.1% Tween 20 (dilution buffer) and incubated overnight at 4 °C. For 1-day experiments, phosphor-p44/42 MAPK (Erk1/2) (Thr202/Tyr204) primary antibody (Cell Signaling Technology) or YAP (Santa Cruz Biotechnology) were diluted 1:200 in dilution buffer and incubated at 4 °C overnight.

Bound antibodies were then fluorescently tagged for 1 h at room temperature with species-appropriate secondary antibodies (for myosin, goat anti-mouse Alexa555 secondary antibody (Molecular Probes); for phosphor-p44/42 MAPK (Erk1/2) (Thr202/Tyr204), goat anti-rabbit Alexa555 (Molecular Probes); for YAP, goat anti-mouse Alexa647 (Abcam)) diluted 1:1000 dilution buffer. Cells were washed three times in 1× PBS after each step. All surfaces were counterstained with NucBlue (Molecular Probes) for 20 min at room temperature to visualize nuclei, and MF20-stained cells were also counterstained with Phalloidin-iFluor 647 (Abcam) to visualize actin. The cells were then imaged at 20× magnification.

### Characterization of Cell Responses

The length of cells transfected with mCherry LifeAct (imaged with TRITC excitation/emission specifications) was measured for the duration of the stimulation to identify cell elongation. For cells transfected with EKAR, the cells were excited with CFP excitation (436 nm), and donor emission (480 nm) and sensitized FRET emission (525 nm emission) were recorded at 0, 10, and 20 min of stimulation to measure ERK phosphorylation. All cell image analyses were conducted using ImageJ.

Surfaces immunostained for pERK and YAP were imaged in stimulated regions (marked by “x”s on the glass slide) and unstimulated regions (>10 mm away from stimulated regions), and average fluorescent intensity of the respective signals were measured in cell nuclei and the cytoplasm. YAP is reported as the ratio of nuclear/cytoplasmic signal.

For cells in the 5-day differentiation experiment, multiple regions of interest were acquired on stimulated and unstimulated surfaces. Cell differentiation was quantified by calculating the percentage of nuclei in a region of interest that were contained within MF20-positive myotubes. Fusion was also quantified as the average number of nuclei per cell. In cases where nuclei were counted, cell area was defined by the f-actin fluorescent signal (and the MF20 signal for differentiation) overlaid with the nuclear signal. Nuclei visualized entirely within a single cell were counted to that cell. The edge of the microscopic field of view was assumed to be the end of the cell.

### Statistics

Statistical analysis was conducted using GraphPad Prism 8 (GraphPad). All experiments were conducted in triplicate. All data are reported as mean ± standard deviation, and statistical significance is determined at α = 0.05 from unpaired, nonparametric analysis of variance (ANOVA) unless otherwise stated, with the exception of data from live-cell 20 min stimulation experiments, which are paired by cell.

## Supporting Information Available

Actuation of OMA-containing hydrogel (Video S1) (AVI)
Actuation of 3D-printed OMA hydrogel (Video S2) (AVI)
Actuation of collagen-OMA hydrogel (Video S3) (AVI)
OMA and hydrogel characterization of OMA containing GelMA, characterization of methacrylated OMAs with HPLC chart, ^1^H NMR and ESI-MS spectra for azido-methacrylate, rheology measurements, NIR responsivity of GelMA with and without OMA, three-dimensional analysis of gel responsivity, effect of laminin in the actuating hydrogel, quantification of local heating on actuating gel and cell viability upon NIR illumination, example of other actuating hydrogels, and differentiation experiment setup (PDF)
Actuation of PEG-OMA hydrogel (Video S4) (AVI)

## Supplementary Material

ab3c00516_si_001.avi

ab3c00516_si_002.avi

ab3c00516_si_003.avi

ab3c00516_si_004.pdf

ab3c00516_si_005.avi

## References

[ref1] Breslin S.; O’Driscoll L. Three-Dimensional Cell Culture: The Missing Link in Drug Discovery. Drug Discovery Today 2013, 18, 240–249. 10.1016/J.DRUDIS.2012.10.003. 23073387

[ref2] Ghallab A. Letter to the Editor: In Vitro Test Systems and Their Limitations. EXCLI J. 2013, 12, 1024–1026. 27034642 PMC4803002

[ref3] Tagle D. A. The NIH Microphysiological Systems Program: Developing in Vitro Tools for Safety and Efficacy in Drug Development. Curr. Opin. Pharmacol. 2019, 48, 146–154. 10.1016/J.COPH.2019.09.007. 31622895

[ref4] Janmey P. A.; Miller R. T. Mechanisms of Mechanical Signaling in Development and Disease. J. Cell Sci. 2011, 124, 9–18. 10.1242/JCS.071001. 21172819 PMC3001405

[ref5] Oria R.; Wiegand T.; Escribano J.; Elosegui-Artola A.; Uriarte J. J.; Moreno-Pulido C.; Platzman I.; Delcanale P.; Albertazzi L.; Navajas D.; Trepat X.; García-Aznar J. M.; Cavalcanti-Adam E. A.; Roca-Cusachs P. Force Loading Explains Spatial Sensing of Ligands by Cells. Nature 2017, 552, 219–224. 10.1038/nature24662. 29211717

[ref6] Majedi F. S.; Hasani-Sadrabadi M. M.; Thauland T. J.; Li S.; Bouchard L. S.; Butte M. J. T-Cell Activation Is Modulated by the 3D Mechanical Microenvironment. Biomaterials 2020, 252, 120058 10.1016/J.BIOMATERIALS.2020.120058. 32413594 PMC7307918

[ref7] Augat P.; Simon U.; Liedert A.; Claes L. Mechanics and Mechano-Biology of Fracture Healing in Normal and Osteoporotic Bone. Osteoporosis Int. 2005, 16, S36–S43. 10.1007/S00198-004-1728-9. 15372141

[ref8] McClure M. J.; Clark N. M.; Hyzy S. L.; Chalfant C. E.; Olivares-Navarrete R.; Boyan B. D.; Schwartz Z. Role of Integrin A7β1 Signaling in Myoblast Differentiation on Aligned Polydioxanone Scaffolds. Acta Biomater. 2016, 39, 44–54. 10.1016/j.actbio.2016.04.046. 27142254

[ref9] Gauthier N. C.; Roca-Cusachs P. Mechanosensing at Integrin-Mediated Cell–Matrix Adhesions: From Molecular to Integrated Mechanisms. Curr. Opin. Cell Biol. 2018, 50, 20–26. 10.1016/J.CEB.2017.12.014. 29438903

[ref10] Wu Y.; Xiang Y.; Fang J.; Li X.; Lin Z.; Dai G.; Yin J.; Wei P.; Zhang D. The Influence of the Stiffness of GelMA Substrate on the Outgrowth of PC12 Cells. Biosci. Rep. 2019, 39, BSR20181748 10.1042/BSR20181748. 30606743 PMC6340955

[ref11] Engler A. J.; Sen S.; Sweeney H. L.; Discher D. E. Matrix Elasticity Directs Stem Cell Lineage Specification. Cell 2006, 126, 677–689. 10.1016/j.cell.2006.06.044. 16923388

[ref12] Engler A. J.; Griffin M. A.; Sen S.; Bönnemann C. G.; Sweeney H. L.; Discher D. E. Myotubes Differentiate Optimally on Substrates with Tissue-like Stiffness: Pathological Implications for Soft or Stiff Microenvironments. J. Cell Biol. 2004, 166, 877–887. 10.1083/jcb.200405004. 15364962 PMC2172122

[ref13] Egusa H.; Kobayashi M.; Matsumoto T.; Sasaki J.-I.; Uraguchi S.; Yatani H. Application of Cyclic Strain for Accelerated Skeletal Myogenic Differentiation of Mouse Bone Marrow-Derived Mesenchymal Stromal Cells with Cell Alignment. Tissue Eng., Part A 2013, 19, 770–782. 10.1089/ten.tea.2012.0164. 23072369

[ref14] Salazar B. H.; Cashion A. T.; Dennis R. G.; Birla R. K. Development of a Cyclic Strain Bioreactor for Mechanical Enhancement and Assessment of Bioengineered Myocardial Constructs. Cardiovasc. Eng. Technol. 2015, 6, 533–545. 10.1007/s13239-015-0236-8. 26577484 PMC4653094

[ref15] Heher P.; Maleiner B.; Prüller J.; Teuschl A. H.; Kollmitzer J.; Monforte X.; Wolbank S.; Redl H.; Rünzler D.; Fuchs C. A Novel Bioreactor for the Generation of Highly Aligned 3D Skeletal Muscle-like Constructs through Orientation of Fibrin via Application of Static Strain. Acta Biomater. 2015, 24, 251–265. 10.1016/j.actbio.2015.06.033. 26141153

[ref16] Andreu I.; Falcones B.; Hurst S.; Chahare N.; Quiroga X.; Le Roux A. L.; Kechagia Z.; Beedle A. E. M.; Elosegui-Artola A.; Trepat X.; Farré R.; Betz T.; Almendros I.; Roca-Cusachs P. The Force Loading Rate Drives Cell Mechanosensing through Both Reinforcement and Cytoskeletal Softening. Nat. Commun. 2021, 12, 4229 10.1038/s41467-021-24383-3. 34244477 PMC8270983

[ref17] Gungordu H. I.; Bao M.; van Helvert S.; Jansen J. A.; Leeuwenburgh S. C. G.; Walboomers X. F. Effect of Mechanical Loading and Substrate Elasticity on the Osteogenic and Adipogenic Differentiation of Mesenchymal Stem Cells. J. Tissue Eng. Regen. Med. 2019, 13, 2279–2290. 10.1002/TERM.2956. 31483956

[ref18] Cui Y.; Hameed F. M.; Yang B.; Lee K.; Pan C. Q.; Park S.; Sheetz M. Cyclic Stretching of Soft Substrates Induces Spreading and Growth. Nat. Commun. 2015, 6, 6333 10.1038/ncomms7333. 25704457 PMC4346610

[ref19] Massai D.; Pisani G.; Isu G.; Rodriguez Ruiz A.; Cerino G.; Galluzzi R.; Pisanu A.; Tonoli A.; Bignardi C.; Audenino A. L.; Marsano A.; Morbiducci U. Bioreactor Platform for Biomimetic Culture and in Situ Monitoring of the Mechanical Response of in Vitro Engineered Models of Cardiac Tissue. Front. Bioeng. Biotechnol. 2020, 8, 733 10.3389/FBIOE.2020.00733. 32766218 PMC7381147

[ref20] Buchmann B.; Fernández P.; Bausch A. R. The Role of Nonlinear Mechanical Properties of Biomimetic Hydrogels for Organoid Growth. Biophys. Rev. 2021, 2, 021401 10.1063/5.0044653. PMC7612859 35722505

[ref21] Aroush D. R.-B.; Barlam D.; Wagner H. D. Generating an Inhomogeneous Stress Field as a Technique to Study Cell Mechanoresponse. Appl. Phys. Lett. 2012, 100, 133703 10.1063/1.3697652.

[ref22] Beroz F.; Jawerth L. M.; Münster S.; Weitz D. A.; Broedersz C. P.; Wingreen N. S. Physical Limits to Biomechanical Sensing in Disordered Fibre Networks. Nat. Commun. 2017, 8, 16096 10.1038/ncomms16096. 28719577 PMC5520107

[ref23] Bhingardive V.; Edri A.; Kossover A.; Le Saux G.; Khand B.; Radinsky O.; Iraqi M.; Porgador A. Nanowire Based Mechanostimulating Platform for Tunable Activation of Natural Killer Cells. Adv. Funct. Mater. 2021, 31, 2103063 10.1002/ADFM.202103063.

[ref24] Tse J. R.; Engler A. J. Stiffness Gradients Mimicking In Vivo Tissue Variation Regulate Mesenchymal Stem Cell Fate. PLoS One 2011, 6, e15978 10.1371/JOURNAL.PONE.0015978. 21246050 PMC3016411

[ref25] Elosegui-Artola A.; Andreu I.; Beedle A. E. M.; Lezamiz A.; Uroz M.; Kosmalska A. J.; Oria R.; Kechagia J. Z.; Rico-Lastres P.; Le Roux A.-L.; Shanahan C. M.; Trepat X.; Navajas D.; Garcia-Manyes S.; Roca-Cusachs P. Force Triggers YAP Nuclear Entry by Regulating Transport across Nuclear Pores. Cell 2017, 171, 1397–1410. 10.1016/j.cell.2017.10.008. 29107331

[ref26] Sutton A.; Shirman T.; Timonen J. V. I.; England G. T.; Kim P.; Kolle M.; Ferrante T.; Zarzar L. D.; Strong E.; Aizenberg J. Photothermally Triggered Actuation of Hybrid Materials as a New Platform for in Vitro Cell Manipulation. Nat. Commun. 2017, 8, 14700 10.1038/ncomms14700. 28287116 PMC5355809

[ref27] Liu Z.; Liu Y.; Chang Y.; Seyf H. R.; Henry A.; Mattheyses A. L.; Yehl K.; Zhang Y.; Huang Z.; Salaita K. Nanoscale Optomechanical Actuators for Controlling Mechanotransduction in Living Cells. Nat. Methods 2016, 13, 143–146. 10.1038/nmeth.3689. 26657558 PMC4732909

[ref28] Ramey-Ward A. N.; Su H.; Salaita K. Mechanical Stimulation of Adhesion Receptors Using Light-Responsive Nanoparticle Actuators Enhances Myogenesis. ACS Appl. Mater. Interfaces 2020, 12, 35903–35917. 10.1021/acsami.0c08871. 32644776 PMC8818098

[ref29] Su H.; Liu Z.; Liu Y.; Ma V. P.-Y.; Blanchard A.; Zhao J.; Galior K.; Dyer R. B.; Salaita K. Light-Responsive Polymer Particles as Force Clamps for the Mechanical Unfolding of Target Molecules. Nano Lett. 2018, 18, 2630–2636. 10.1021/ACS.NANOLETT.8B00459. 29589759 PMC6110664

[ref30] Zhao J.; Su H.; Vansuch G. E.; Liu Z.; Salaita K.; Dyer R. B. Localized Nanoscale Heating Leads to Ultrafast Hydrogel Volume-Phase Transition. ACS Nano 2019, 13, 515–525. 10.1021/acsnano.8b07150. 30574782 PMC6467806

[ref31] Yang J.; Hazlett L.; Landauer A. K.; Franck C. Augmented Lagrangian Digital Volume Correlation (ALDVC). Exp. Mech. 2020, 60, 1205–1223. 10.1007/S11340-020-00607-3.

[ref32] Chen T.-H.; Chen C.-Y.; Wen H.-C.; Chang C.-C.; Wang H.-D.; Chuu C.-P.; Chang C.-H. YAP Promotes Myogenic Differentiation via the MEK5-ERK5 Pathway. FASEB J. 2017, 31, 2963–2972. 10.1096/fj.201601090R. 28356344

[ref33] Knight J. D.; Kothary R. The Myogenic Kinome: Protein Kinases Critical to Mammalian Skeletal Myogenesis. Skeletal Muscle 2011, 1, 29 10.1186/2044-5040-1-29. 21902831 PMC3180440

[ref34] Fanning P. J.; Emkey G.; Smith R.; Grodzinsky A.; Szasz N.; Trippel S. Mechanical Regulation of Mitogen-Activated Protein Kinase Signaling in Articular Cartilage. J. Biol. Chem. 2003, 278, 50940–50948. 10.1074/JBC.M305107200. 12952976

[ref35] Harvey C. D.; Ehrhardt A.; Cellurale C.; Zhong H.; Yasuda R.; Davis R.; Svoboda K. A Genetically Encoded Fluorescent Sensor of ERK Activity. Proc. Natl. Acad. Sci. U.S.A. 2008, 105, 19264–19269. 10.1073/PNAS.0804598105. 19033456 PMC2614750

[ref36] Mangner N.; Linke A.; Oberbach A.; Kullnick Y.; Gielen S.; Sandri M.; Hoellriegel R.; Matsumoto Y.; Schuler G.; Adams V. Exercise Training Prevents TNF-α Induced Loss of Force in the Diaphragm of Mice. PLoS One 2013, 8, e52274 10.1371/JOURNAL.PONE.0052274. 23300968 PMC3534708

[ref37] Lee Y.; Lee J. M.; Bae P. K.; Chung I. Y.; Chung B. H.; Chung B. G. Photo-Crosslinkable Hydrogel-Based 3D Microfluidic Culture Device. Electrophoresis 2015, 36, 994–1001. 10.1002/elps.201400465. 25641332

[ref38] Heltmann-Meyer S.; Steiner D.; Müller C.; Schneidereit D.; Friedrich O.; Salehi S.; Engel F. B.; Arkudas A.; Horch R. E. Gelatin Methacryloyl Is a Slow Degrading Material Allowing Vascularization and Long-Term Use in Vivo. Biomed. Mater. 2021, 16, 065004 10.1088/1748-605X/ac1e9d. 34406979

[ref39] Lim J. W.; Kim H.; Kim Y.; Shin S. G.; Cho S.; Jung W. G.; Jeong J. H. An Active and Soft Hydrogel Actuator to Stimulate Live Cell Clusters by Self-Folding. Polymers 2020, 12, 583 10.3390/polym12030583. 32150989 PMC7182895

[ref40] Yang G. H.; Kim W.; Kim J.; Kim G. A Skeleton Muscle Model Using GelMA-Based Cell-Aligned Bioink Processed with an Electric-Field Assisted 3D/4D Bioprinting. Theranostics 2021, 11, 48–63. 10.7150/THNO.50794. 33391460 PMC7681100

[ref41] Kim C.; Young J. L.; Holle A. W.; Jeong K.; Major L. G.; Jeong J. H.; Aman Z. M.; Han D.-W.; Hwang Y.; Spatz J. P.; Choi Y. S. Stem Cell Mechanosensation on Gelatin Methacryloyl (GelMA) Stiffness Gradient Hydrogels. Ann. Biomed. Eng. 2020, 48, 893–902. 10.1007/S10439-019-02428-5. 31802282

[ref42] Costantini M.; Testa S.; Fornetti E.; Barbetta A.; Trombetta M.; Cannata S. M.; Gargioli C.; Rainer A. Engineering Muscle Networks in 3D Gelatin Methacryloyl Hydrogels: Influence of Mechanical Stiffness and Geometrical Confinement. Front Bioeng. Biotechnol. 2017, 5, 22 10.3389/FBIOE.2017.00022. 28439516 PMC5383707

[ref43] Yamada N.; Okano T.; Sakai H.; Karikusa F.; Sawasaki Y.; Sakurai Y. Thermo-responsive Polymeric Surfaces; Control of Attachment and Detachment of Cultured Cells. Makromol. Chem., Rapid Commun. 1990, 11, 571–576. 10.1002/marc.1990.030111109.

[ref44] Chen Y. X.; Cain B.; Soman P. Gelatin Methacrylate-Alginate Hydrogel with Tunable Viscoelastic Properties. AIMS Mater. Sci. 2017, 4, 363–369. 10.3934/MATERSCI.2017.2.363.

[ref45] Csapo R.; Gumpenberger M.; Wessner B. Skeletal Muscle Extracellular Matrix – What Do We Know About Its Composition, Regulation, and Physiological Roles? A Narrative Review. Front. Physiol. 2020, 11, 253 10.3389/FPHYS.2020.00253. 32265741 PMC7096581

[ref46] Pęziński M.; Daszczuk P.; Pradhan B. S.; Lochmüller H.; Prószyński T. J. An Improved Method for Culturing Myotubes on Laminins for the Robust Clustering of Postsynaptic Machinery. Sci. Rep. 2020, 10, 4524 10.1038/s41598-020-61347-x. 32161296 PMC7066178

[ref47] Denes L. T.; Riley L. A.; Mijares J. R.; Arboleda J. D.; McKee K.; Esser K. A.; Wang E. T. Culturing C2C12 Myotubes on Micromolded Gelatin Hydrogels Accelerates Myotube Maturation. Skeletal Muscle 2019, 9, 17 10.1186/s13395-019-0203-4. 31174599 PMC6555731

[ref48] Senf S. M. Skeletal Muscle Heat Shock Protein 70: Diverse Functions and Therapeutic Potential for Wasting Disorders. Front. Physiol. 2013, 4, 330 10.3389/FPHYS.2013.00330. 24273516 PMC3822288

[ref49] Purschke M.; Laubach H. J.; Anderson R. R.; Manstein D. Thermal Injury Causes DNA Damage and Lethality in Unheated Surrounding Cells: Active Thermal Bystander Effect. J. Invest. Dermatol. 2010, 130, 86–92. 10.1038/JID.2009.205. 19587691

[ref50] Soleilhac A.; Girod M.; Dugourd P.; Burdin B.; Parvole J.; Dugas P.-Y.; Bayard F.; Lacôte E.; Bourgeat-Lami E.; Antoine R. Temperature Response of Rhodamine B-Doped Latex Particles. From Solution to Single Particles. Langmuir 2016, 32, 4052–4058. 10.1021/ACS.LANGMUIR.6B00647. 27042942

[ref51] Hayat H.; Friedberg I. Heat-Induced Alterations in Cell Membrane Permeability and Cell Inactivation of Transformed Mouse Fibroblasts. Int. J. Hyperthermia 1986, 2, 369–378. 10.3109/02656738609004967. 3805806

[ref52] Charonis A. S.; Tsilibary E. C.; Yurchenco P. D.; Furthmayr H. Binding of Laminin to Type IV Collagen: A Morphological Study. J. Cell Biol. 1985, 100, 1848–1853. 10.1083/jcb.100.6.1848. 3997977 PMC2113590

[ref53] Gribova V.; Liu C. Y.; Nishiguchi A.; Matsusaki M.; Boudou T.; Picart C.; Akashi M. Construction and Myogenic Differentiation of 3D Myoblast Tissues Fabricated by Fibronectin-Gelatin Nanofilm Coating. Biochem. Biophys. Res. Commun. 2016, 474, 515–521. 10.1016/J.BBRC.2016.04.130. 27125461 PMC5024749

[ref54] Belkin A. M.; Zhidkova N. I.; Balzac F.; Altruda F.; Tomatis D.; Maier A.; Tarone G.; Koteliansky V. E.; Burridge K. Beta 1D Integrin Displaces the Beta 1A Isoform in Striated Muscles: Localization at Junctional Structures and Signaling Potential in Nonmuscle Cells. J. Cell Biol. 1996, 132, 211–226. 10.1083/JCB.132.1.211. 8567725 PMC2120711

[ref55] McClure M. J.; Ramey A. N.; Rashid M.; Boyan B. D.; Schwartz Z. Integrin-A7 Signaling Regulates Connexin 43, M-Cadherin, and Myoblast Fusion. Am. J. Physiol. Cell Physiol. 2019, 316, C876–C887. 10.1152/ajpcell.00282.2018. 30892939

[ref56] Zhang H.; Pasolli H. A.; Fuchs E. Yes-Associated Protein (YAP) Transcriptional Coactivator Functions in Balancing Growth and Differentiation in Skin. Proc. Natl. Acad. Sci. U.S.A. 2011, 108, 2270–2275. 10.1073/PNAS.1019603108. 21262812 PMC3038759

[ref57] Wang Y.; Dong Q.; Zhang Q.; Li Z.; Wang E.; Qiu X. Overexpression of Yes-Associated Protein Contributes to Progression and Poor Prognosis of Non-Small-Cell Lung Cancer. Cancer Sci. 2010, 101, 1279–1285. 10.1111/J.1349-7006.2010.01511.X. 20219076 PMC11158334

[ref58] Michailovici I.; Harrington H. A.; Azogui H. H.; Yahalom-Ronen Y.; Plotnikov A.; Ching S.; Stumpf M. P. H.; Klein O. D.; Seger R.; Tzahor E. Nuclear to Cytoplasmic Shuttling of ERK Promotes Differentiation of Muscle Stem/Progenitor Cells. Development 2014, 141, 2611 10.1242/DEV.107078. 24924195 PMC4067960

[ref59] Yun M. H.; Gates P. B.; Brockes J. P. Sustained ERK Activation Underlies Reprogramming in Regeneration-Competent Salamander Cells and Distinguishes Them from Their Mammalian Counterparts. Stem Cell Rep. 2014, 3, 15–23. 10.1016/J.STEMCR.2014.05.009. PMC4110794 25068118

[ref60] De Larichaudy J.; Zufferli A.; Serra F.; Isidori A. M.; Naro F.; Dessalle K.; Desgeorges M.; Piraud M.; Cheillan D.; Vidal H.; Lefai E.; Némoz G. TNF-α- and Tumor-Induced Skeletal Muscle Atrophy Involves Sphingolipid Metabolism. Skeletal Muscle 2012, 2, 2 10.1186/2044-5040-2-2. 22257771 PMC3344678

[ref61] Aurora A.; Garg K.; Corona B. T.; Walters T. J. Physical Rehabilitation Improves Muscle Function Following Volumetric Muscle Loss Injury. BMC Sports Sci. Med. Rehabil. 2014, 6, 41 10.1186/2052-1847-6-41. 25598983 PMC4297368

[ref62] Liu J.; Saul D.; Böker K. O.; Ernst J.; Lehman W.; Schilling A. F. Current Methods for Skeletal Muscle Tissue Repair and Regeneration. BioMed Res. Int. 2018, 2018, 1984879 10.1155/2018/1984879. 29850487 PMC5926523

[ref63] Rooney J. E.; Welser J. V.; Dechert M. A.; Flintoff-Dye N. L.; Kaufman S. J.; Burkin D. J. Severe Muscular Dystrophy in Mice That Lack Dystrophin and Alpha7 Integrin. J. Cell Sci. 2006, 119, 2185–2195. 10.1242/jcs.02952. 16684813

[ref64] Chali F.; Desseille C.; Houdebine L.; Benoit E.; Rouquet T.; Bariohay B.; Lopes P.; Branchu J.; Della Gaspera B.; Pariset C.; Chanoine C.; Charbonnier F.; Biondi O. Long-Term Exercise-Specific Neuroprotection in Spinal Muscular Atrophy-like Mice. J. Physiol. 2016, 594, 1931–1952. 10.1113/JP271361. 26915343 PMC4818605

[ref65] Pennisi C. P.; Olesen C. G.; de Zee M.; Rasmussen J.; Zachar V. Uniaxial Cyclic Strain Drives Assembly and Differentiation of Skeletal Myocytes. Tissue Eng., Part A 2011, 17, 2543–2550. 10.1089/ten.tea.2011.0089. 21609183

[ref66] Liu F.; Lagares D.; Choi K.; Stopfer L.; Marinković A.; Vrbanac V.; Probst C.; Hiemer S.; Sisson T.; Horowitz J.; Rosas I.; Fredenburgh L.; Feghali-Bostwick C.; X V.; Tager A.; Tschumperlin D. Mechanosignaling through YAP and TAZ Drives Fibroblast Activation and Fibrosis. Am. J. Physiol. Lung Cell Mol. Physiol. 2015, 308, L344–L357. 10.1152/AJPLUNG.00300.2014. 25502501 PMC4329470

[ref67] Knoll S. G.; Ali M. Y.; Saif M. T. A. A Novel Method for Localizing Reporter Fluorescent Beads Near the Cell Culture Surface for Traction Force Microscopy. J. Vis. Exp. 2014, 91, e51873 10.3791/51873. PMC4828080 25286326

[ref68] Franck C.; Hong S.; Maskarinec S. A.; Tirrell D. A.; Ravichandran G. Three-Dimensional Full-Field Measurements of Large Deformations in Soft Materials Using Confocal Microscopy and Digital Volume Correlation. Exp. Mech. 2007, 47, 427–438. 10.1007/S11340-007-9037-9.

[ref69] Mercer S. E.; Ewton D. Z.; Deng X.; Lim S.; Mazur T. R.; Friedman E. Mirk/Dyrk1B Mediates Survival during the Differentiation of C2C12 Myoblasts. J. Biol. Chem. 2005, 280, 25788–25801. 10.1074/jbc.M413594200. 15851482 PMC1201501

[ref70] Bader D.; Masaki T.; Fischman D. A. Immunochemical Analysis of Myosin Heavy Chain during Avian Myogenesis in Vivo and in Vitro. J. Cell Biol. 1982, 95, 763–770. 10.1083/jcb.95.3.763. 6185504 PMC2112936

