# Supplementary material for: Optomechanically Actuated Hydrogel Platform for Cell Stimulation with Spatial and Temporal Resolution
Source: ACS Biomater Sci Eng. 2023 Aug 21;9(9):5361–75. doi: 10.1021/acsbiomaterials.3c00516 (PMC10498418; doi:10.1021/acsbiomaterials.3c00516)
Supplement: Supplementary file 4 — ab3c00516_si_004.pdf [file ab3c00516_si_004.pdf]

Supporting Information:

An Optomechanically Actuated Hydrogel Platform for Cell Stimulation with Spatial and Temporal Resolution

Allison N. Ramey-Ward 1, Yixiao Dong 2, Jin Yang 3, Hiroaki Ogasawara 2, Elizabeth C. Bremer-Sai 3, Olga Brazhkina 1, Christian Franck 3, Michael Davis 1, Khalid Salaita* 1,2

1 Wallace H. Coulter Department of Biomedical Engineering, Georgia Institute of Technology & Emory University, Atlanta, GA 30322, USA

2. Department of Chemistry, Emory University, Atlanta, GA 30322, USA

3. Department of Mechanical Engineering, University of Wisconsin – Madison, Madison, WI 53706, USA

*Corresponding Author: k.salaita@emory.edu

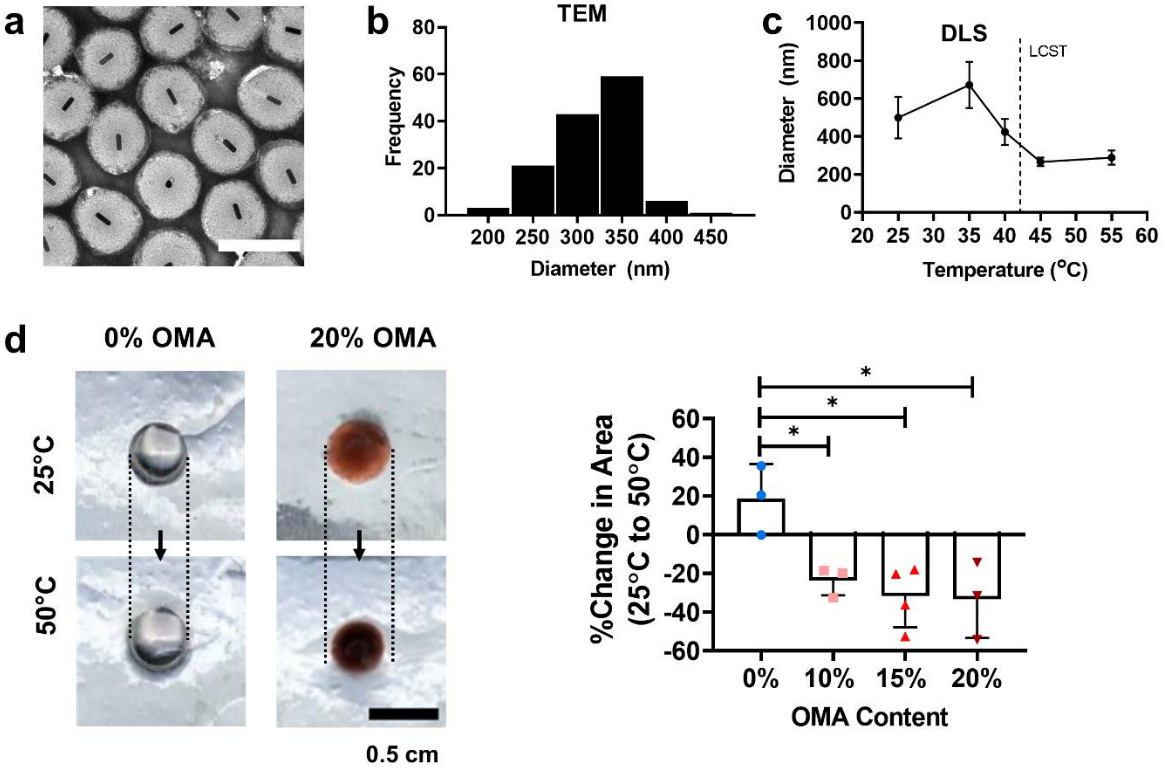

Figure S1: OMA and Hydrogel Characterization. a) Transmission electron microscopy (TEM) imaging showing OMA size and AuNR incorporation. Scale bar = 0.5 µm. b) Quantification of monodispersity in OMA diameter by TEM, showing most particles have a dry diameter of 300-400 nm. c) Temperature-dependent dynamic light scattering showing average hydrodynamic diameter ± standard deviation from n = 3 independent preparations of OMAs. The understood lower critical solution temperature (LCST) of NIPMAm, 42°C, is marked by the dotted line. d) (Left) Photographs of bulk hydrogels +/- OMAs before and after heating to the indicated temperature. Red coloration is due to AuNR content. Scale bar: 0.5 cm. (Right) Change in measured area of hydrogels after heating. As expected, plot shows that % change in area is OMA content-dependent. *: $p<0.05$, **: $p<0.01$ by one-way analysis of variance (ANOVA) with Tukey's multiple comparisons post-test for n=3 independently prepared hydrogels.

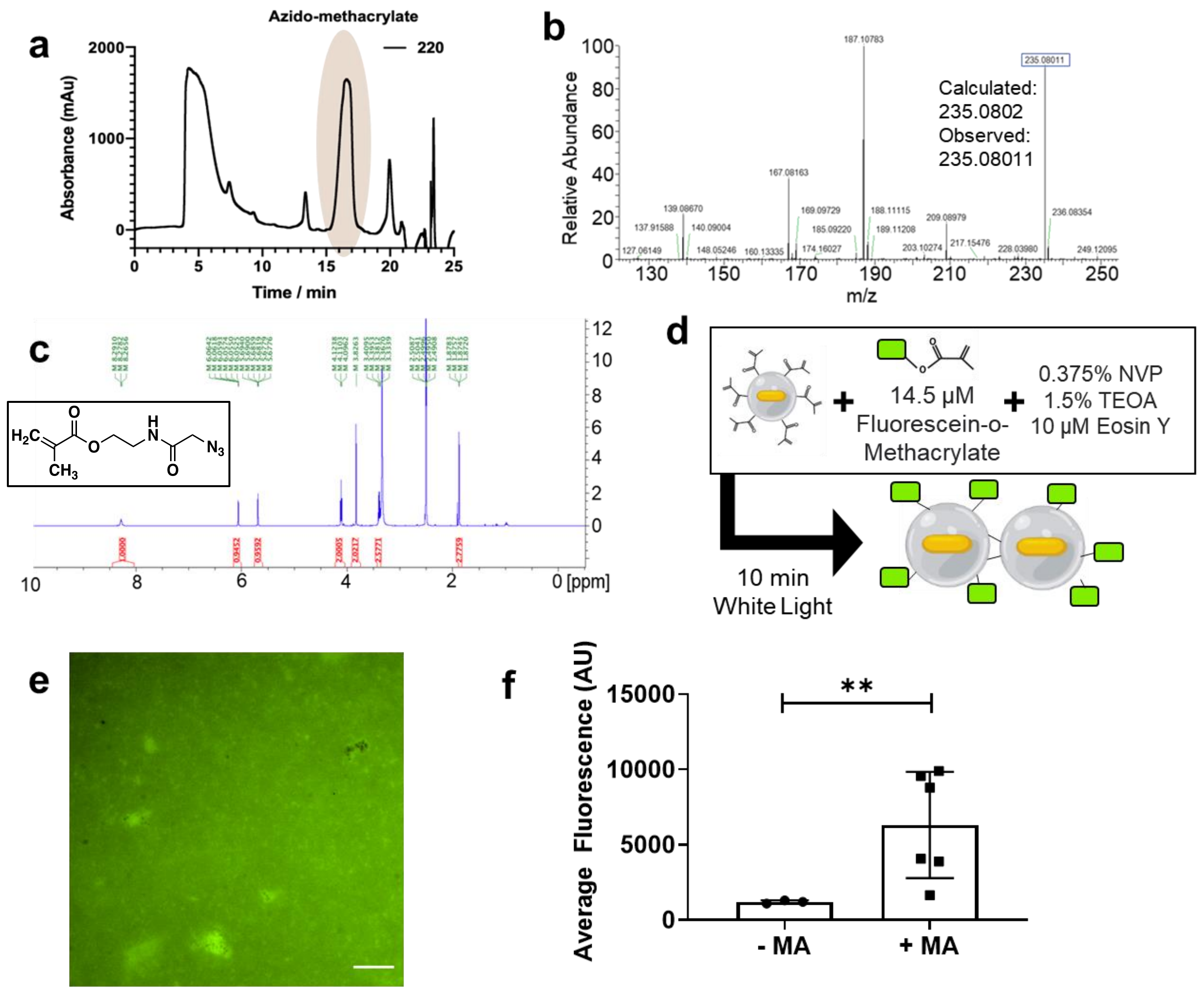

Figure S2: Development and Characterization of Methacrylated OMAs. a) HPLC trace of 220 nm absorbance showing isolation of peak at approximately 16 minutes (brown circle) as the product. b) ESI mass spectrum shows a product peak of 235.08011, aligning well with the calculated mass of the expected molecule of 235.0802. c) Nuclear magnetic resonance verification of isolated product, with characteristic peaks in the spectrum correlated to molecule features. d) Schematic showing labeling of OMAs with fluorescein-O-methacrylate to measure incorporation of methacrylate groups onto OMA surfaces. e) Representative fluorescent image of methacrylated OMAs incubated in solution in crosslinking conditions with fluorescein-O-methacrylate to verify methacrylate incorporation. Scale bar = 5 µm. f) Graph quantifying average fluorescence (± standard deviation) of fluorescein-O-methacrylate on OMAs in solution. **: $p<0.01$ by Mann-Whitney test on n= 3 independent OMA preparations.

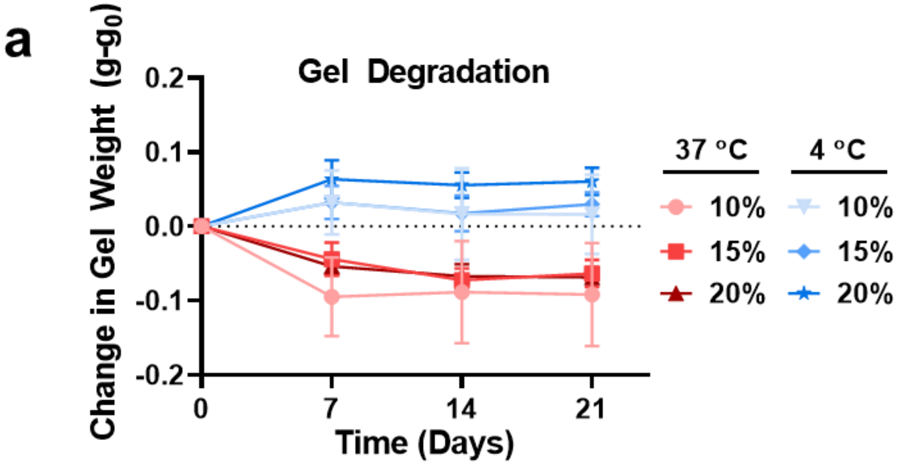

Figure S3: OMA Hydrogel Degradation. a) Graph showing average change in gel weight in grams (± standard deviation) in n=3 actuating hydrogels with varying OMA content incubated at 37°C or 4°C. for 3 weeks. Average gel starting weight was 94 mg.

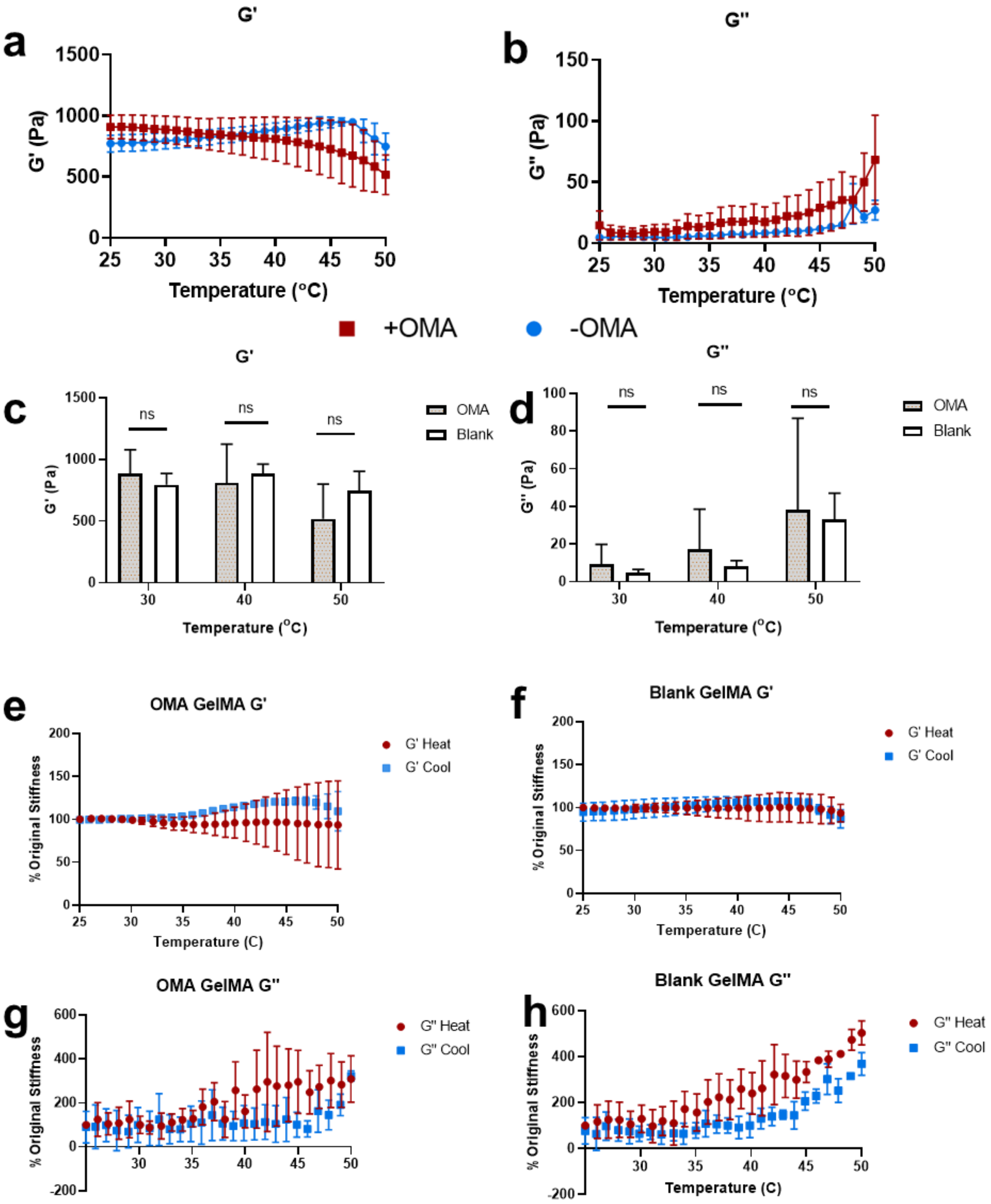

Figure S4: Further Characterization of Gel Rheology. a-b) Temperature-controlled rheometry showing mean ± standard deviation G' (a) and G" (b) for n = 3 independently prepared gel samples. c-d) Graphs quantifying G' (c) and G" (d), showing no differences in response between control GelMA hydrogels and OMA containing gels. By mixed effects model with Sidak's multiple comparisons on n=3 independently prepared hydrogels. e-h) Temperature-controlled

rheometry showing mean ± standard deviation heating and cooling curves of n=3 independently prepared hydrogel samples, demonstrating a slight difference in heating and cooling behavior at higher temperatures in G' for OMA containing gels (e) and G" in GelMA with and without OMAs (g-h), but not in G' of GelMA without OMAs (f).

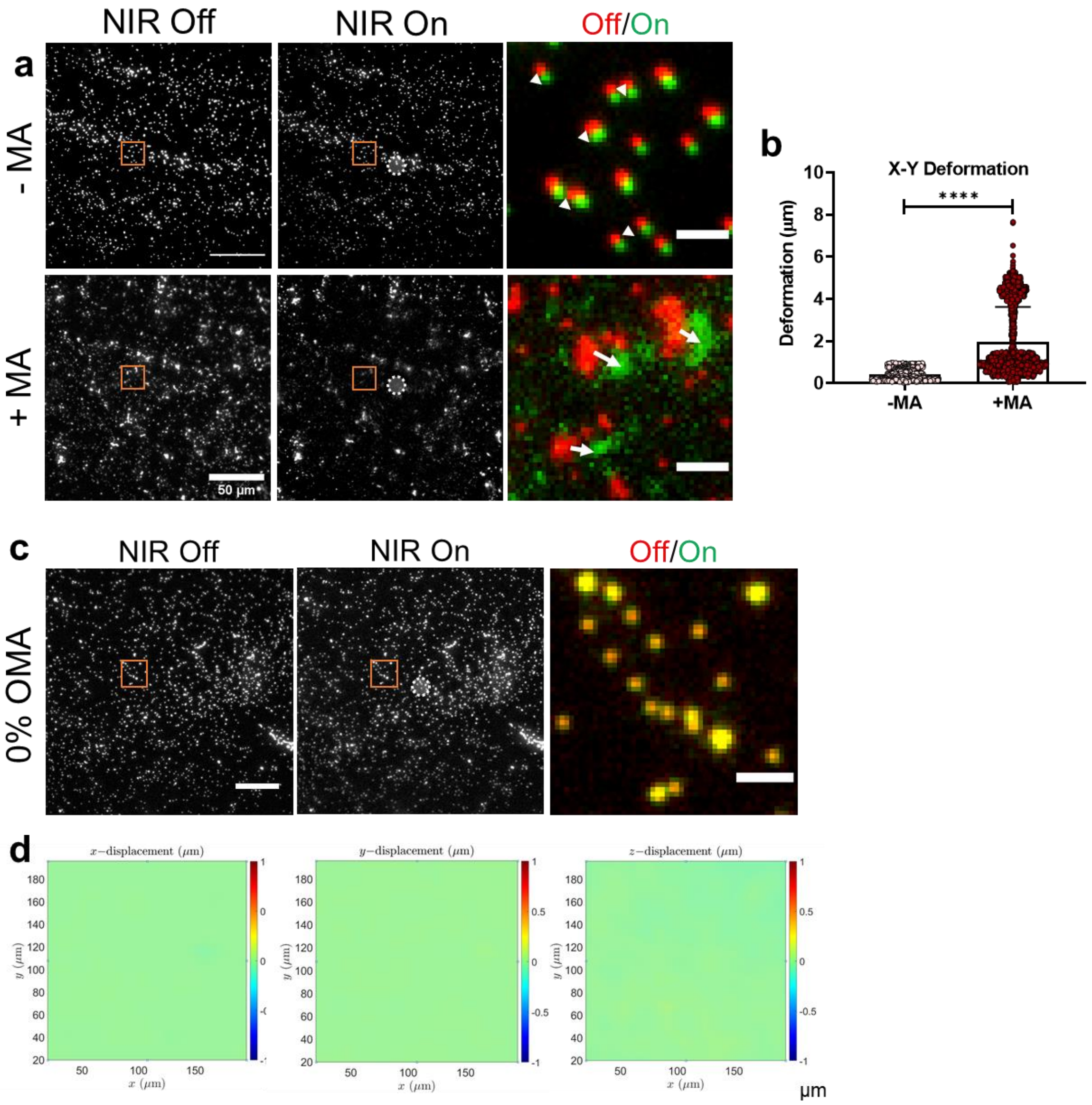

Figure S5: NIR Responsivity of GelMA With and Without OMAs.

a) Fluorescent images showing movement of fluorescent beads on the surface of hydrogels containing 20% non-methacrylated OMAs (top) and OMAs modified with azidomethacrylate (bottom) without NIR stimulation (left) and with NIR stimulation (33.1 μW/μm^2 power density, middle) at the region indicated by the white circle. Scale bar = 50 μm. Orange boxes denote outset of fluorescent beads showing power-dependent deformation (right). Scale bar = 5 μm. Bead movement direction from NIR off (red) to NIR on (green) indicated with white arrows. b) Graph quantifying average gel deformation ± standard deviation in gels with methacrylated and non-methacrylated OMAs. ****: $p<0.0001$ by Mann-Whitney test for all tracked beads in n=2 (-MA) and n=3 (+MA) independently prepared hydrogels. c) Representative image of fluorescent bead displacement with NIR laser off (left) or on at 33.1 μW/μm^2 power density at the location of the white circle (middle). Scale bar = 50 μm. Orange boxes indicate outset image, showing an

overlay of particles in the NIR off (red) and NIR on (green). The lack of separation of red and green particles indicates no motion of the gel. d) Gel deformation in the x direction (left), y direction (middle) and z direction (right) as calculated by ALDVC shows no bead movement between NIR off and on states, demonstrating actuation is a function of OMA content.

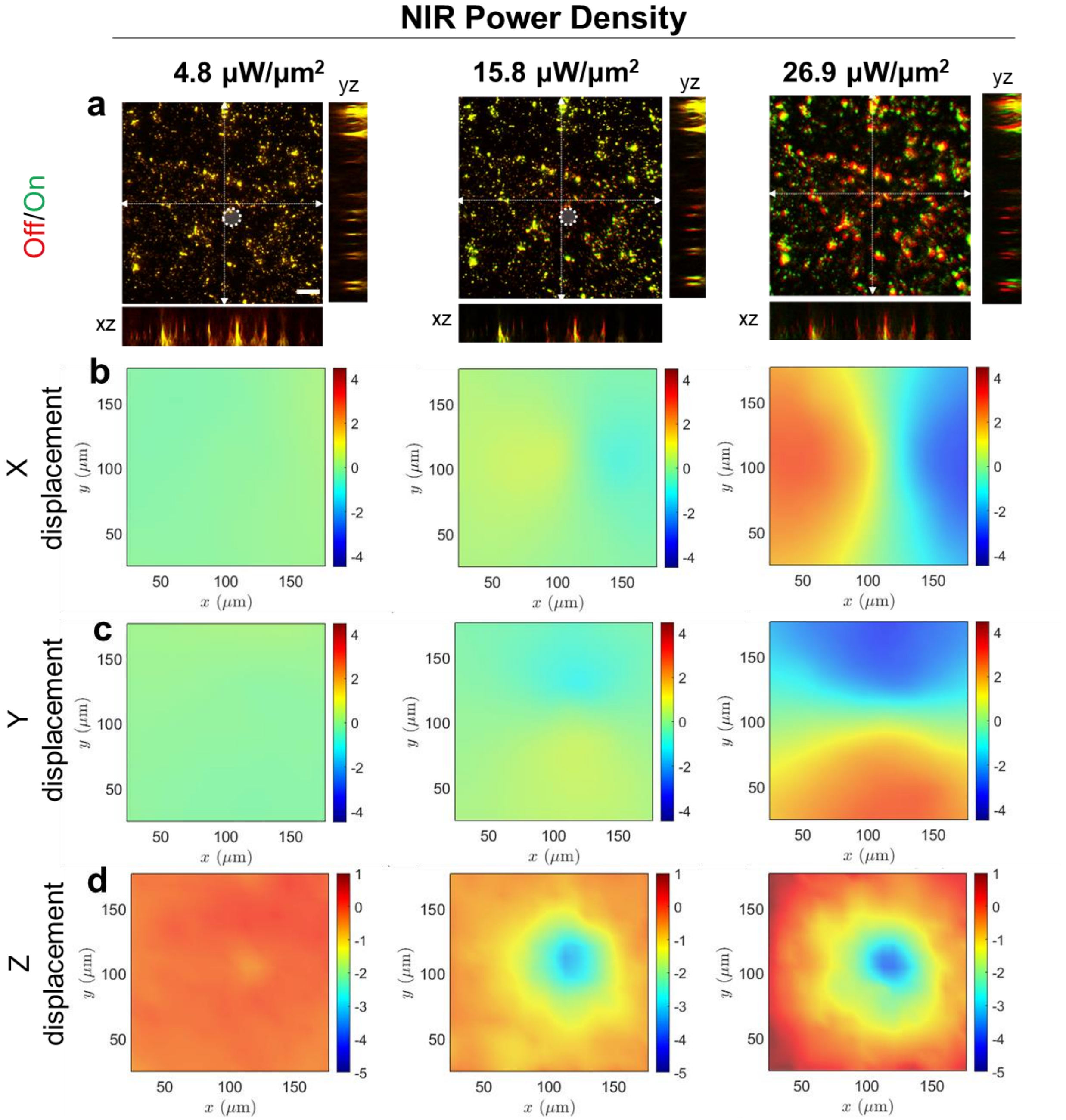

Figure S6: Three-Dimensional Analysis of Gel Responsivity to NIR Illumination Power. a) Gels containing 20% OMAs were stimulated with NIR light at varying power densities in the location of the white circle. Representative images of fluorescent beads on the surface of the gel were taken in the laser off (red) and laser on (green) conditions and overlayed to show gel movement in the XY plane. Scale bar = 50 μm. Additionally, orthogonal images were rendered from 40 μm z-stacks along the regions marked with white double arrows to show deformation in XZ and YZ planes with illumination off (red) or on (green). Augmented Lagrangian digital volume correlation (ALDVC) shows gel deformation in the plane of the image (the XY plane, b-c), as well as in the z dimension (d) of an example gel, showing increasing magnitude of response in all dimensions as a function of increasing OMA content.

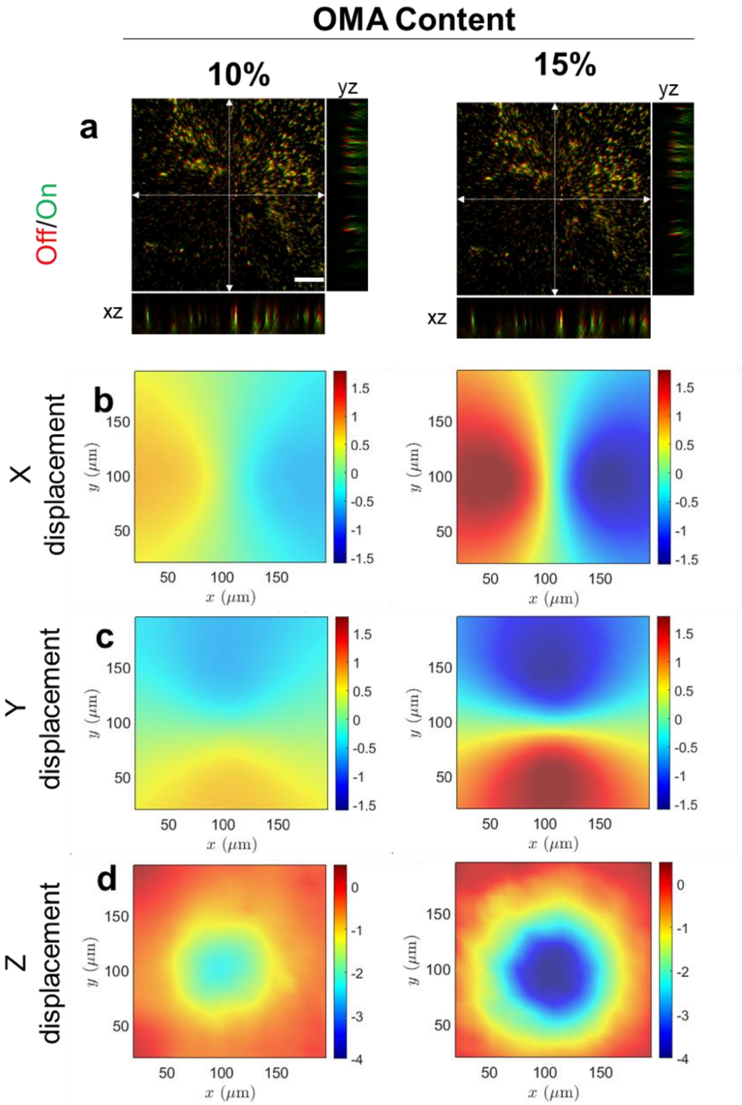

Figure S7: Three-Dimensional Analysis of Gel Responsiveness to OMA Content. a) Gels containing varying concentrations of OMAs were stimulated with NIR light at a power density of 33.1 μW/μm^2 in the location of the white circle. Representative images of fluorescent beads on the surface of the gel were taken in the laser off (red) and laser on (green) conditions and overlayed to show gel movement in the XY plane. Scale bar = 50 μm. Additionally, orthogonal images were rendered from 40 μm z-stacks along the regions marked with white double arrows to show deformation in XZ and YZ planes with illumination off (red) or on (green). Augmented Lagrangian digital volume correlation (ALDVC) shows gel deformation in the plane of the image (the XY plane, b-c), as well as in the z dimension (d) of an example gel, showing increasing magnitude of response in all dimensions as a function of increasing OMA content.

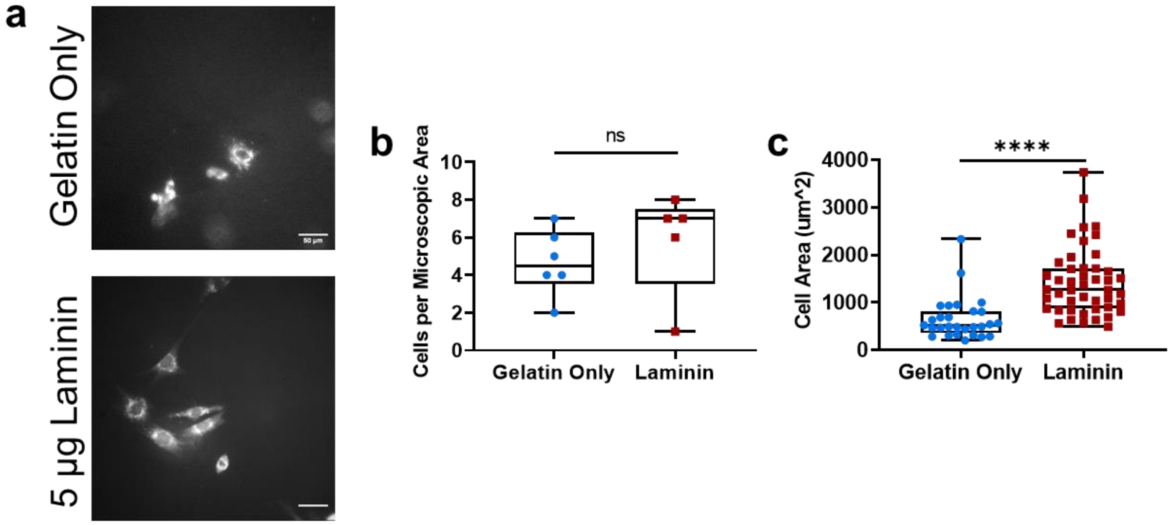

Figure S8: Effect of Laminin in Actuating Hydrogels on Cell Adhesion. a) Representative images of C2C12 myoblasts stained with CellTracker CMTMR plated onto actuating hydrogels (20% OMA content) without (top) or with (bottom) mouse laminin. Scale bar = 50 μm. b) Box plot quantifying range of cells per field of view after 18 hours in culture on actuating hydrogels with and without laminin show no significant difference by a Mann-Whitney test on 6 fields of view from n = 3 independently prepared hydrogels. c) Box plot quantifying range of cell spreading area after 18 hours in culture on actuating hydrogels with and without laminin. ****: $p<0.0001$ by a Mann-Whitney test on at least 30 cells each from n = 3 independently prepared hydrogels.

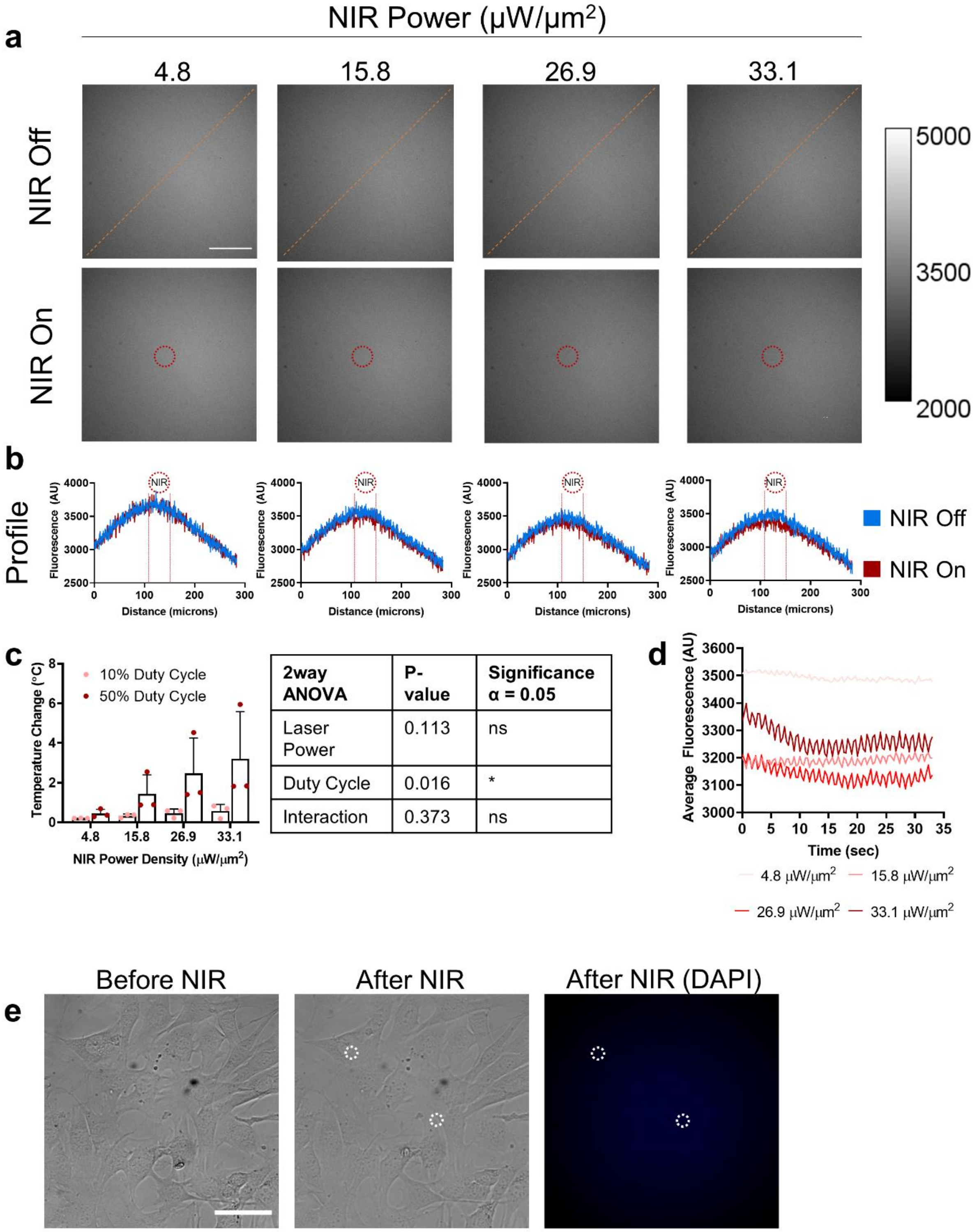

2way ANOVA	P-value	Significance α = 0.05
Laser Power	0.113	ns
Duty Cycle	0.016	*
Interaction	0.373	ns

Figure S9: Quantification of Local Heating on Actuating Gel Surfaces. a) Representative images of rhodamine solution fluorescence at the surface of actuating hydrogels without NIR illumination (top) or with NIR illumination of varying intensities (bottom). Scale bar = 50 μm. Red

circle indicates NIR illumination location. Orange line indicates area of line scan fluorescence profile. b) Profiles of fluorescence across each image, demonstrating the illumination profile of the field and the difference in fluorescence with (red) and without (blue) NIR illumination. Red circle and lines indicate area of NIR illumination. c) Graph showing change in rhodamine fluorescence when gels are exposed to NIR illumination at 1 Hz at varying laser powers and duty cycles(10% = 100 ms on time, 50% = 500 ms on time) as a metric of local heating on the gel surface. Bars show mean ± standard deviation from n=3 independently prepared hydrogel samples each with 20% OMA content. A two-way analysis of variance shows laser on time but not power is responsible for increased heating. d) Representative trace of sample fluorescence at varying laser powers (50% duty cycle), showing periodic signal change corresponding to the 1 Hz stimulation. e) Representative images of C2C12 myoblasts incubated with DAPI before (left) and after (middle) NIR stimulation (white circles). No DAPI signal was observed (right) after NIR stimulation. Scale bar = 50 μm

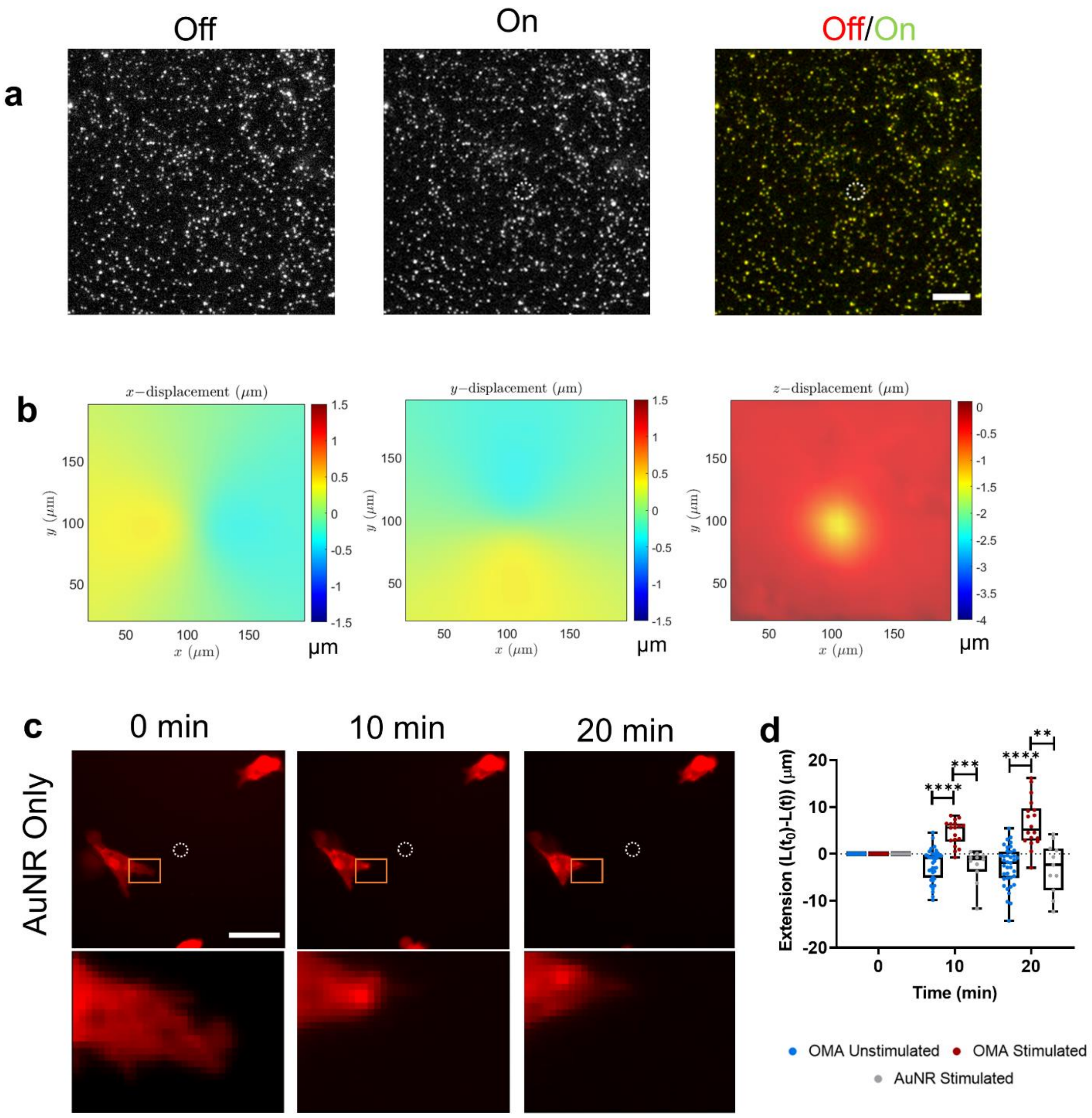

Figure S10: Responsivity of Gels Containing AuNRs. a) Representative images of deformation without (left, and red) and with (middle, and green) NIR stimulation at 33.1 μW/μm^2 (white circle) of gels containing either 20% OMAs (bottom) or the same volume of AuNRs (top). Scale bar = 20 μm. b) Deformation gradients within gels containing only OMAs, showing a small amount of deformation in the x (left), y (middle), and z (right) dimensions. c) Representative images of mCherry LifeAct-transfected C2C12 myoblasts exposed to NIR stimulation (1 Hz, 500 ms on time, 33.1 μW/μm^2) for 20 minutes. Orange box represents outset region. Scale bar = 50 μm. d) Box plot quantifying range of extension of cell edges in the direction of NIR stimulus for unstimulated and stimulated hydrogels containing 20% OMAs (data from main text Figure 3) for

n = 3 independently prepared hydrogel samples. **: $p<0.01$, ***: $p<0.001$, ****: $p<0.0001$ by two-way analysis of variance with Tukey's multiple comparisons.

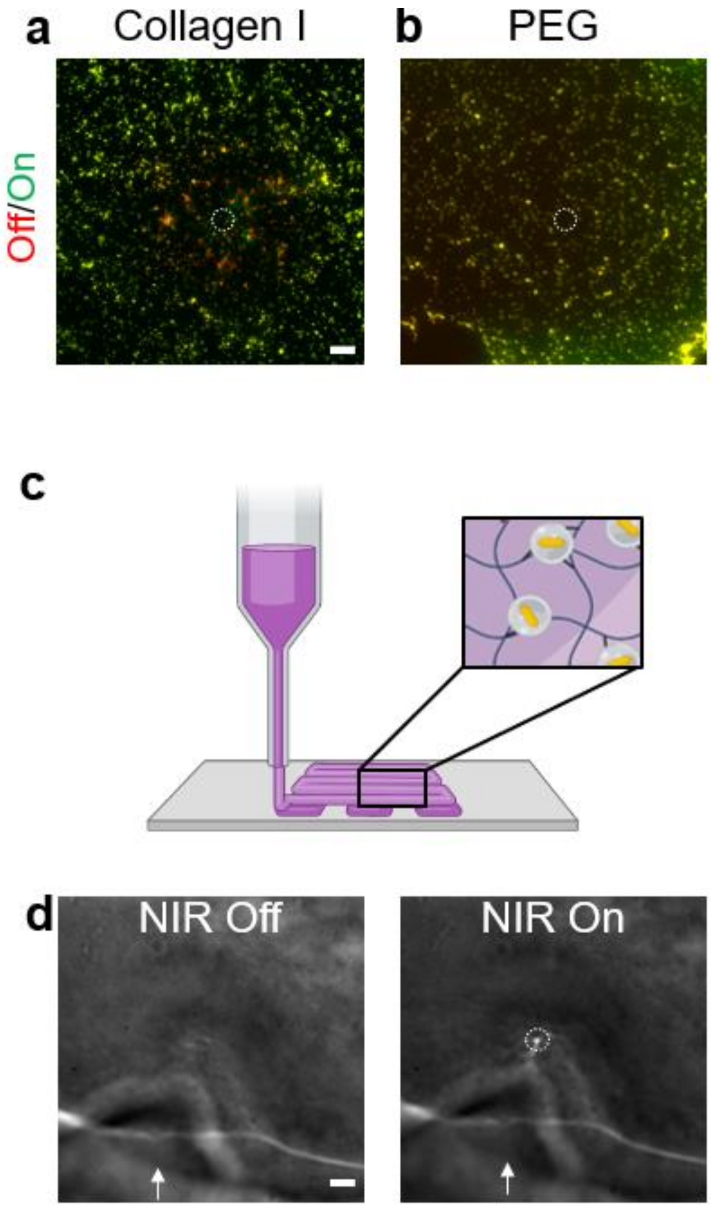

Figure S11 - Other Actuating Hydrogels.

a-b) Fluorescent images showing movement of fluorescent beads on the surface of hydrogels comprised of a) rat tail Collagen I and b) PEG containing 20% OMAs without NIR stimulation (off, red) and with 48 mW NIR stimulation (on, green) at the region indicated by the white circle, demonstrating the ability of this method to be applied to multiple hydrogel systems. Scale bar: 20 μm. c) Schematic diagram of 3D printing of OMA-containing GelMA hydrogels. d) Brightfield images showing response of 3D printed hydrogels containing 20% OMAs without (left) and with (right) 48 mW NIR illumination (white circle). The edge of the hydrogel is indicated with the white arrow. Scale bar: 20 μm.

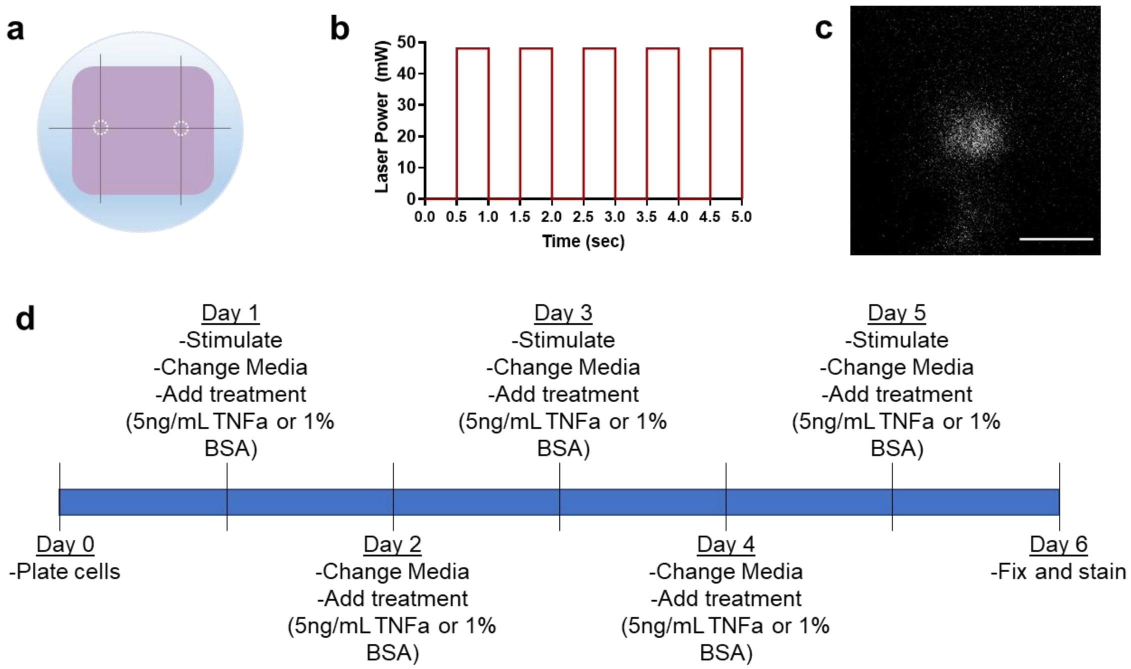

Figure S12: Differentiation Experiment Setup. a) Schematic drawing of hydrogel sample (purple) on a glass slide (blue), showing the guidemarks (gray) scratched on the back of the glass slide to guide repeated NIR stimulation (white circle) and subsequent imaging in the same area over the course of multi-day experiments. b) Representative graph of NIR stimulation used in this work. For cell experiments in this work, NIR laser illumination is a square function with a frequency of 1 Hz, duty cycle of 50%, and power of 33.1 $\mu W/\mu m^2$. c) Bleed-through of 785 nm NIR laser imaged with high exposure in 670 nm emission channel (Cy5). Scale bar = 50 μm. d) Timeline of treatment in myogenesis studies. Surfaces were NIR stimulated every other day, and media was refreshed with treatment condition (TNFα or vehicle control).

Supplemental Video 1: Actuation of OMA-Containing Hydrogel. Fluorescent beads on the surface of actuating hydrogels were imaged while stimulated at 1 Hz with 33.1 μW/μm^2 NIR power density, the conditions used for experiments in this work. Scale bar = 50 μm.

Supplemental Video 2: Actuation of 3D Printed OMA Hydrogels. 3D printed GelMA hydrogel constructs were stimulated at 1 Hz with 33.1 μW/μm^2 NIR power density. Scale bar = 100 μm.

Supplemental Video 3: Actuation of Collagen-OMA Hydrogels. Fluorescent beads on the surface of collagen 1 hydrogels containing OMAs were imaged while stimulated at 1 Hz with 33.1 μW/μm^2 NIR power density. Scale bar = 50 μm.

Supplemental Video 4: Actuation of PEG-OMA Hydrogels. Fluorescent beads on the surface of PEG hydrogels containing OMAs were imaged while stimulated at 1 Hz with 33.1 μW/μm^2 NIR power density. Scale bar = 50 μm.
